# A Practical Guide for Using Electrochemical Dilatometry as Operando Tool in Battery and Supercapacitor Research

**DOI:** 10.1002/ente.202101120

**Published:** 2022-03-10

**Authors:** Ines Escher, Matthias Hahn, Guillermo A. Ferrero, Philipp Adelhelm

**Affiliations:** ^1^ Institut für Chemie Humboldt Universität zu Berlin 12489 Berlin Germany; ^2^ R&D EL-Cell GmbH 21079 Hamburg Germany; ^3^ Helmholtz-Zentrum Berlin Joint Research Group Operando Battery Analysis (CE-GOBA) 14109 Berlin Germany

**Keywords:** batteries, electrochemical dilatometry, electrodes, operando measurements, supercapacitors

## Abstract

Lithium‐ion batteries and related battery concepts show an expansion and shrinkage (“breathing”) of the electrodes during cell cycling. The dimensional changes of an individual electrode or a complete cell can be continuously measured by electrochemical dilatometry (ECD). The obtained data provides information on the electrode/cell reaction itself but can be also used to study side reactions or other relevant aspects, e.g., how the breathing is influenced by the electrode binder and porosity. The method spans over a wide measurement range and allows the determination of macroscopic as well as nanoscopic changes. It has also been applied to supercapacitors. The method has been developed already in the 1970s but recent advancements and the availability of commercial setups have led to an increasing interest in ECD. At the same time, there is no “best practice” on how to evaluate the data and several pitfalls exist that can complicate the comparison of literature data. This review highlights the recent development and future trends of ECD and its use in battery and supercapacitor research. A practical guide on how to evaluate the data is provided along with a discussion on various factors that influence the measurement results.

## Graphical abstract

Electrochemical dilatometry is a powerful operando technique to measure the thickness change in an electrochemical device during cycling. Several studies have been published in recent years, investigating batteries and supercapacitors. This review presents selected examples from literature, a guideline on how to evaluate dilatometry data, and gives information on which effects have an influence on the data.

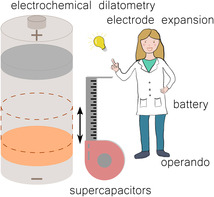

## 1 Introduction

Dilatometry is a classical technique in materials science that has been used since decades to measure the expansion or shrinkage of a macroscopic sample while changing another experimental parameter. Typically, this is done as a function of temperature, which allows to determine the thermal expansion coefficient of a material.^[^ ^1^ ^]^ It can be also used to determine vacancy concentrations of a material, as shown in the classical experiment by Simmons and Balluffi for aluminum in 1960.^[^ ^2^ ^]^ Another possibility of applying dilatometry is to follow the progress of chemical reactions. In the 1970s, the method was also used in electrochemical experiments, the dilatation of a sample is measured as a function of the electrode potential (or cell voltage). In a pioneering work, Metrot et al. applied this “electrochemical dilatometry (ECD)” to determine the thickness change of a graphite electrode upon intercalation with a boron complex.^[^ ^3^ ^]^ The method is often mentioned as in situ method but the mode of operation is actually operando, that is, the dilatation is recorded continuously. A particular advantage of dilatometry is its wide measurement range, macroscopic changes as well as changes down to the nanometer regime can be detected, depending on the setup. Despite this advantage, the overall number of studies on ECD was limited for a long time. This is because the experimental setups were typically self‐made and a lot of expertise was required to obtain reliable results where, for example, cell leakage or data drift (e.g., due to temperature changes) complicated the analysis.

In the past years, however, the situation changed and the ECD technique saw a strong revival. While commercial devices became available, it was also realized that the method can provide valuable information on batteries and supercapacitors. The popular lithium‐ion batteries (LIBs)^[^ ^4^ ^]^ as well as alternative concepts such as sodium‐ion batteries (SIBs)^[^ ^5^ ^]^ rely on intercalation and conversion reactions. Insertion of ions into electrodes leads to changes in the crystal lattice and the formation of new phases and, as a result, also to a macroscopic change of the electrode. This macroscopic change can be quite different from the crystal lattice change because the electrode is a porous particle network rather than a single crystal. Large changes are generally undesired as they lead to particle cracking and loss of contact and thus compromise the cycle life of the battery. Silicon, for example, is a very attractive anode material for LIBs as its theoretical capacity is ten times larger than the one of graphite, today's commercially most commonly used material. The problem with using pure Si as electrode is that lithiation results in a volume expansion of several hundred percent which leads to particle cracking and poor cycle life.^[^ ^6^ ^]^ Graphite, on the other hand, only expands by around 10% when being lithiated. Commercial LIB anode materials therefore contain at most only few wt% of Si that is dispersed in a graphite matrix.^[^ ^7^ ^]^ This way, the expansion and shrinkage of the electrode during lithiation/delithiation (also called “breathing”) is still tolerable. ECD provides direct and quantitative information on the degree of breathing and is therefore an important tool to optimize such electrodes.

**Figure 1:**
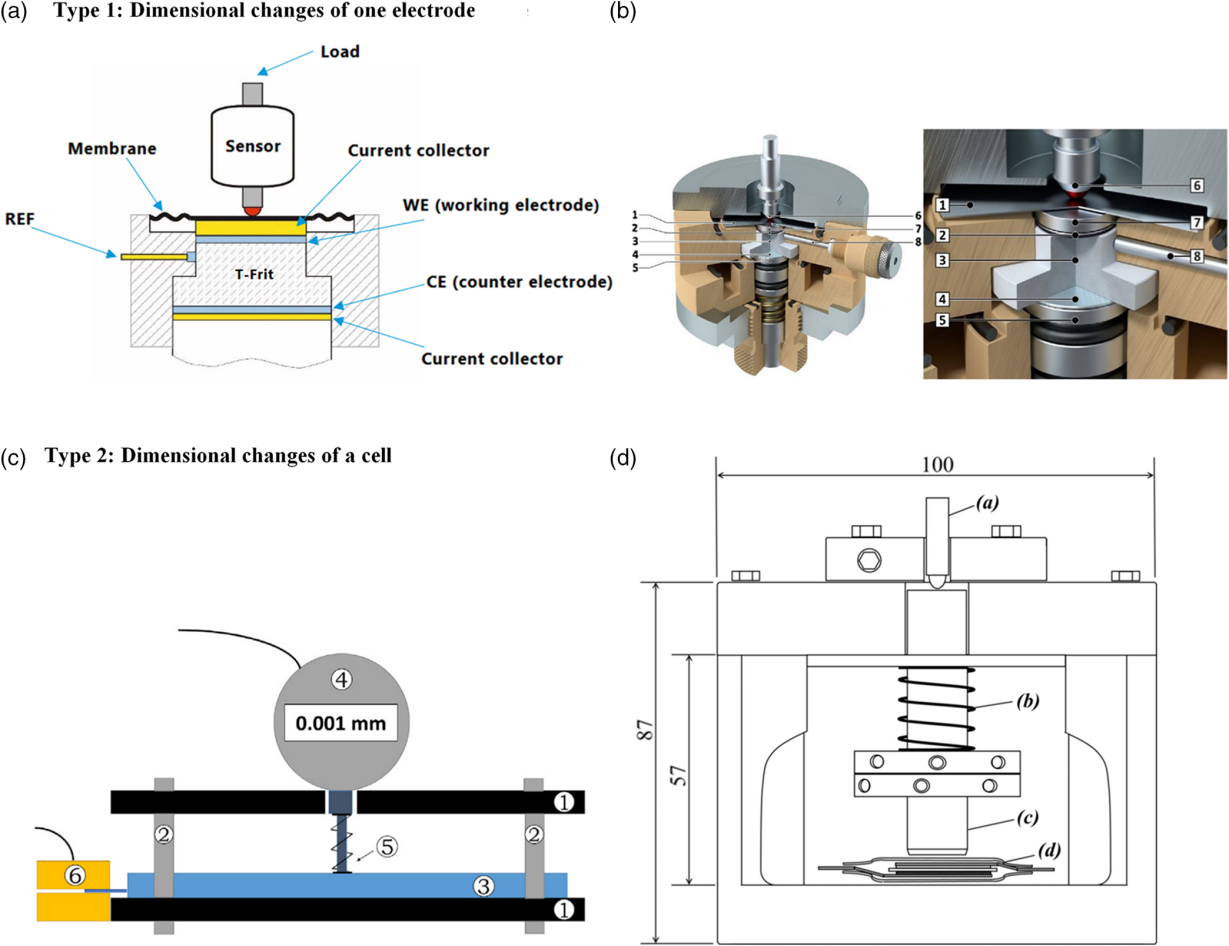
Two different dilatometer setups. Type 1: Thickness change of the working electrode is recorded, solely. This is implemented through the use of a stiff separator. a) A detailed description of the commercial ECD‐3 nanosetup from EL‐Cell^[8o]^ as well as b) visualization of this setup. Reproduced with permission.^[8c]^ Copyright 2017, Elsevier Ltd. Type 2: Thickness change of the whole cell (including working electrode, counter electrode, separators, and housing) is measured. c) Measurement setup using an Al plate (1), threaded rod (2), cell (3), dial gauge (4), a tip with spring and metallic plate (1 cm^2^) (5), and electric contact pads (gold plated). (6) Reproduced with permission.^[8e]^ Copyright 2016, Elsevier B.V. d) Schematic illustration using a) a displacement transducer, b) a spring, c) a spindle, and d) a flexible bag. Reproduced with permission.^[^ ^18^ ^]^ Copyright 2014, The Electrochemical Society. The image in (a) was reproduced with permission from EL‐Cell GmbH.

At the same time, not only the breathing of the electrodes but also of the whole cell is relevant for battery application. Two different types of ECD setups therefore exist, as shown in **Figure** **1**. Type 1 allows the determination the thickness change of one electrode (the working electrode). This is done using a stiff glass separator to ensure that only the changes on this electrode are measured.^[^ ^8^ ^]^ This configuration is most suited for scientific experiments as the thickness change can be related to distinct electrode processes. Much more information than “just” the electrode thickness change can be obtained from these measurements. Type 2 setups are used to determine the thickness change of a whole electrochemical cell. The signal therefore includes changes of both electrodes and other cell components and provides information on more applied aspects of batteries.^8^, ^9^ Mostly pouch cells are used for this purpose.

This review focuses on the application of ECD to battery and supercapacitor electrodes as well as practical aspects of evaluating ECD data. In Section 2, the ECD method is introduced by discussing some examples on battery and supercapacitor electrodes. In Section 3, the data evaluation and different processes observed in ECD data will be discussed. The fourth section deals with the challenges related to ECD as well as future developments.

## 2 ECD for Electrochemical Energy Storage Devices

The thickness of battery and supercapacitor electrodes is typically in the range of 30–100 μm. A characteristic feature of modern ECD devices is that it combines a high resolution (≤ 5 nm) with a wide measurement range (up to 250 μm). This means that battery electrode reactions (thickness changes in the micrometer range) can be followed as well as film formation (formation of solid–electrolyte interphases [SEIs]) or adsorption processes (supercapacitors).

### 2.1 Electrode Reactions in Batteries

Most ECD studies on batteries have been done on LIBs. Different cathode materials (layered oxides,^9^, ^10^ graphite [anion intercalation],^[8c,8f]^ as well as anode materials [graphite,^8^, ^9^, ^11^ transition metal oxides,^8^, ^12^ alloy‐based materials]^[^ ^13^ ^]^) have been investigated with this method. In addition, many studies were done on silicon‐based materials^8^, ^9^, ^14^ as their large volume expansion is a known issue. Different methods to reduce this volume change were therefore explored, including different binder materials,^8^, ^14^, ^15^ covering the electrodes with a thin film,^[14b,14c]^ choosing different slurry pH values for the preparation,^[14e]^ using different conductive additives,^[14f]^ combining silicon with other elements,^8^, ^14^ and fabricating specialized nanostructures^[14l]^. Different full‐cell configurations (graphite/ lithium nickel manganese cobalt oxides [NMC],^8^, ^9^, ^16^ graphite/ lithium cobalt oxide [LCO],^9^, ^17^ graphite/ LiMn_2_O_4_,^[17b]^ lithium–titanate–oxide/NMC,^[^ ^18^ ^]^ silicon–graphene/NMC,^[9h]^ graphite‐blended Si/LCO,^[9k]^ graphite‐blended Si/C/LCO,^[9n]^ and graphite‐blended SiN(P)/NMC^[9m]^) have been also studied using ECD.

**Figure 2:**
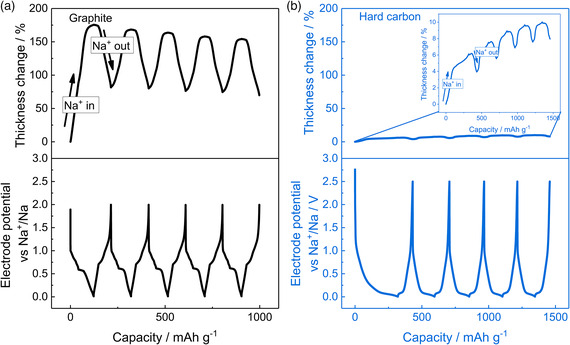
A comparison of ECD results for Na storage in graphite and hard carbon electrodes. The graphs show the change in electrode potential and electrode thickness over five cycles at constant current for a) a graphite electrode upon intercalation/deintercalation of sodium ions along with solvent molecules (solvent co‐intercalation) and b) a hard carbon electrode upon intercalation/deintercalation of sodium ions. The co‐intercalation of solvent molecules in case of (a) leads to a much larger electrode breathing compared with case (b). a) Adapted under terms of the CC‐BY license.^[20b]^ Copyright 2021, the Authors. Published by Wiley‐VCH GmbH. b) Adapted under terms of the CC‐BY license.^[20j]^ Copyright 2021, the Authors. Published by Wiley‐VCH GmbH. Sodium is used as counter and reference electrode.

ECD has been recently also applied to sodium‐ion batteries that may complement LIBs in the near future. While the energy density of SIBs is slightly lower compared with LIBs, the main aim is to develop batteries based on abundant materials that lead to lower costs and less supply risks.^[^ 5, 19 ^]^ Several electrode materials for SIBs have been investigated with ECD.^[^ ^20^ ^]^ Graphite turns out to be a very useful study case for ECD studies, as large and highly reversible volume changes can occur during cycling in ether (glyme) electrolytes.^[20b,20f,20h,20i]^ Using these electrolytes, the intercalation of sodium ions into the graphite lattice includes co‐intercalation of glyme molecules which leads to a large lattice expansion. Upon sodiation, the graphene layer distance increases more than threefold from 0.335 nm to about 1.2 nm.^[^ ^21^ ^]^ The reaction is highly reversible and hence the electrode breathing during cycling can be easily followed by ECD. The influence of various experimental conditions such as the electrolyte composition or the type of binder has been studied by ECD for this reaction.^[20b,20f,20h,20i]^ An illustrative example is shown in **Figure** **2**a), which shows results of a graphite electrode over five charge/discharge cycles. The solvent co‐intercalation leads to an electrode thickness change in the range of 50–175% during cycling.^[20b]^ Hard carbon electrodes have been also studied for which the volume change is notably smaller and typically less than 5%^[20c,20j]^ (see Figure 2b). This directly indicates that solvent co‐intercalation does not take place, which can be ascribed to the much more rigid structure of hard carbon compared with graphite.

**Figure 3:**
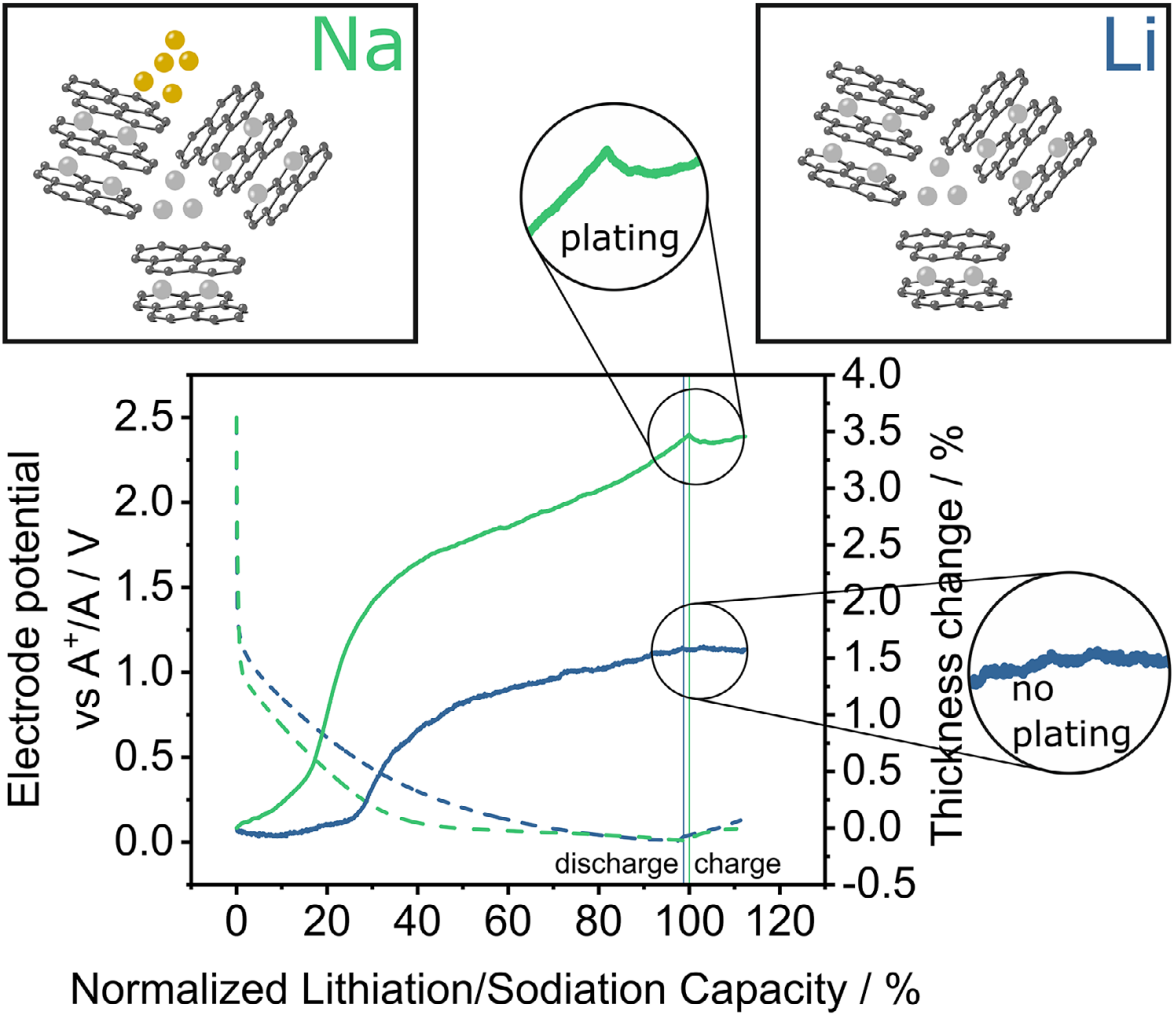
A comparison of ECD results for sodium and lithium storage in hard carbon at constant current. The ECD signal reveals differences in the charge storage mechanism at low potentials (plating vs no plating). Dashed lines indicate the electrode potentials and full lines indicate the dilatation. Reproduced under terms of the CC‐BY license.^[20j]^ Copyright 2021, the Authors. Published by Wiley‐VCH GmbH.

**Figure** **3** shows a comparison for lithium and sodium storage in hard carbon. At low voltage, the ECD data show that the storage mechanism for lithium and sodium in hard carbon may be different. The sudden increase in electrode thickness at low redox potentials in case of sodium is likely associated with plating of sodium metal and shows that the ECD method can be used to study charge storage mechanisms.^[20j]^

Other active materials such as tin–carbon composites,^[20d]^ tin–antimony composite materials,^[20g]^ and tin–phosphide materials^[20a,20k]^ have been also investigated by this technique.

**Figure 4:**
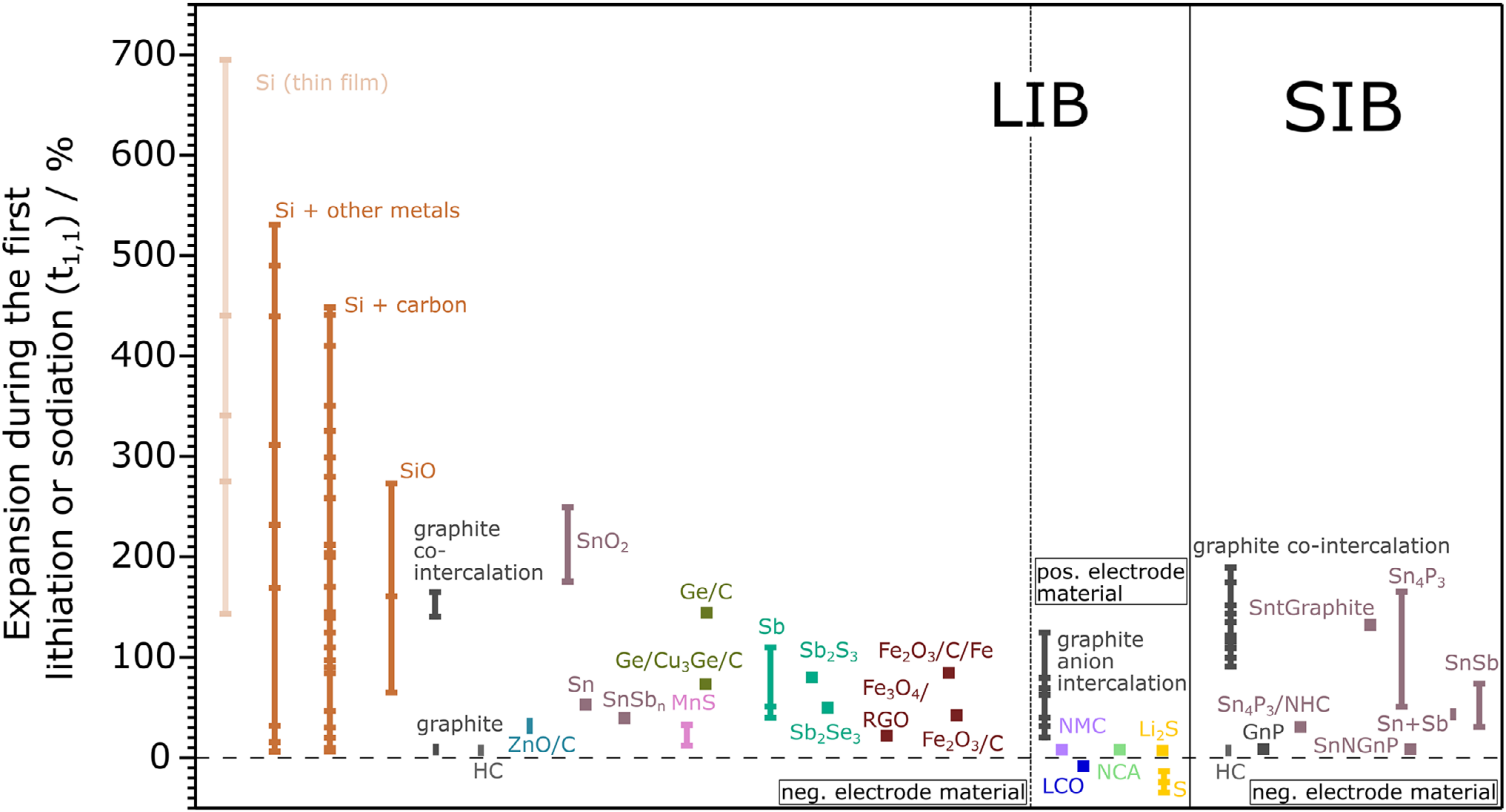
A comparison of different materials used for LIBs and SIBs and their thickness change of the electrode in the first cycle (*t*_1,1_) measured by ECD. Lithium cobalt oxide (LCO), lithium nickel manganese cobalt oxides (NMC), lithium nickel cobalt aluminum oxide (NCA), hard carbon (HC), Sn–graphite composite material (SntGraphite), Sn–P composite combined with nitrogen‐doped hard carbon (Sn_4_P_3_/NHC), Sn at nitrogen‐doped graphite nanoplatelets (SnNGnP), and graphene nanoplatelets (GnP). Data are grouped by color depending on different types of active materials. Markers on the line represent electrode expansions that have been found in different experiments. For small variations, a square shape represents a single experiment, whereas a rectangular shape represents multiple experiments that resulted in similar results. Extreme variations for one material result from the variety of studies performed on some active materials and secondary factors having an influence on the result (see Chapter 4). Data from refs. [8, 9, 10, 11, 12, 13‐14, 15, 20, 22] (assignment of the different references to the materials can be found in Table S1 and S2, Supporting Information).

**Figure** **4** shows an overview of the thickness expansion during the first lithiation or sodiation (*t*_1,1_) for different electrode materials, used in LIBs as well as SIBs. Only references where the expansion of the electrode alone was measured were chosen for this graph. As shown, most studies were performed on negative‐electrode materials for which the expansion is typically much larger compared with positive‐electrode materials. Note that a comparison of negative and positive electrode materials is complicated by the fact that the starting point for the measurement of the former is usually the delithiated/desodiated state (cycling starts with the lithiation/sodiation), while it is the opposite for the latter. In case of full cells, this labeling is difficult as the terms (de‐)lithiated cannot be applied, because both negative and positive electrodes are matters of interest. Therefore, a more precise description is needed in this case. In Figure 4, the delithiated state is used as the starting value for negative as well as positive electrode materials. (for simplicity, we refer to lithiation/delithiation though of course the same nomenclature can be applied for other ions [Na, K, Mg,…]). Assignments of the materials to the references are given in Table S1 and S2, Supporting Information.

In addition, ECD has also been used to investigate the volume expansion in lithium sulfur cells. Herein, a collapse of the electrode thickness from 13% to 37% has been found in the first discharge due to the dissolution of sulfur in the organic electrolyte.^[22h]^ In another study, Li_2_S on activated carbon (AC) has been used; herein, an expansion of ≈0.5% during first lithiation has been found, which is far below the usual expansion of ≈80% when sulfur is converted to Li_2_S.^[22g]^ For a more comprehensive summary on ECD studies of battery electrodes, the reader is referred to a review recently published by Michael et al.^[^ ^23^ ^]^

### 2.2 Full Cells

Determining the thickness change of electrodes is particularly important from a practical point of view because a battery should not show a thickness increase of more than 5% during cycling.^[^ ^24^ ^]^ As the expansion of the electrodes can be tailored by adjusting their porosity or composition, ECD studies on electrodes and the complete cell can be helpful to reach such goals. When charging LIBs or SIBs, the negative electrode generally expands (storing lithium or sodium), whereas the positive electrode shrinks (releasing lithium or sodium). By careful design, the volume changes of both electrodes during cycling may (partially) compensate each other, minimizing the total changes on the cell level. Note, however, that common LIB‐layered positive electrode materials like LCO, NMC, or lithium nickel cobalt aluminum oxides (NCA) show an (partial) electrode increase during delithiation.^9^, ^22^, ^25^ This is because lithium extraction from the layered structures leads to an increasing coulomb repulsion of the layers.^[22a]^ In full‐cell ECD setups, the challenge of measuring the change of two electrodes simultaneously can be mitigated using lithium titanate (LTO) as the zero‐strain material.^9^, ^11^, ^16^, ^18^ It was found that the expansion coefficient of LTO is three times smaller than NMC and 30 times smaller than graphite.^9^, ^26^

### 2.3 Side Reactions and SEI Formation

**Figure 5:**
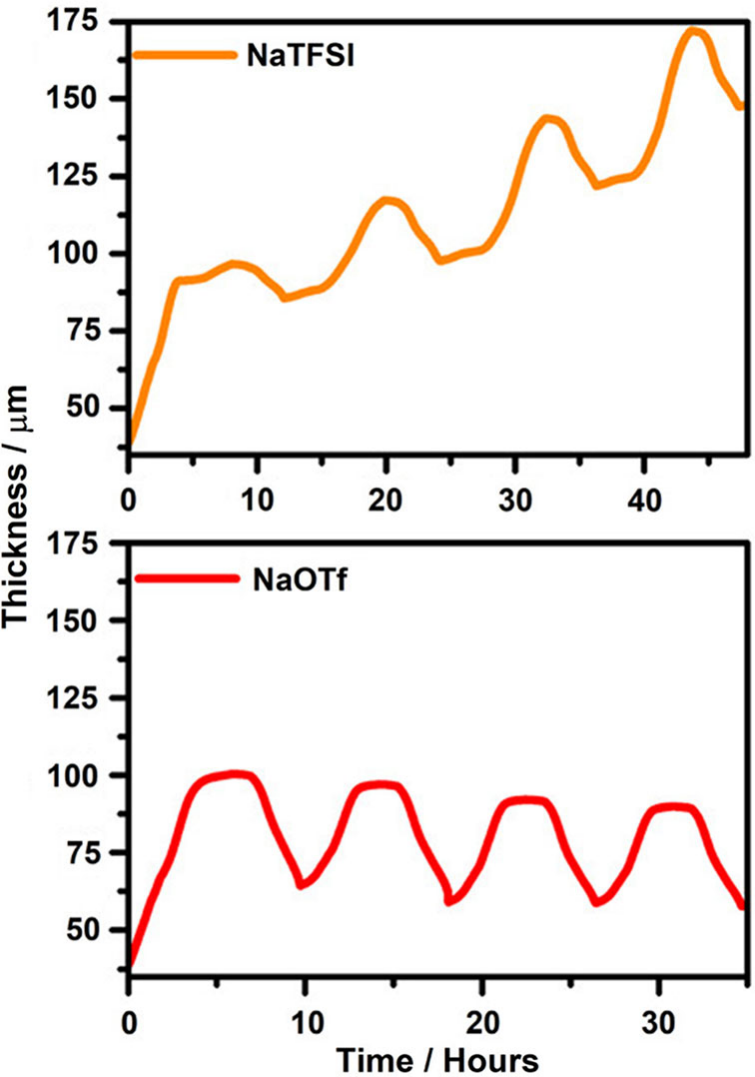
Examples of ECD and side reactions. The graphs compare the thickness change of graphite electrodes over four cycles at constant current upon intercalation/deintercalation of sodium ions along with solvent molecules. The solvent in both cases is diglyme, while the conductive salt is either NaTFSI or NaOTf. A continuous growth of the electrode thickness due to side reactions is found in case of NaTFSI. Adapted with permission.^[20i]^ Copyright 2019, American Chemical Society.

Next to actual storage mechanisms (ion intercalation, ion adsorption), ECD also provides information on side reactions that may occur at electrodes. An example is the intercalation of ithium ions into graphite from propylene carbonate (PC)‐based electrolytes. PC shows very desirable properties as the electrolyte solvent but it co‐intercalates into the graphite lattice along with lithium ions. The co‐intercalation of PC is highly undesirable as the process is irreversible and leads to delamination of the graphite lattice and hence structural degradation of the electrode. This can be seen from a large thickness expansion and unstable signals.^[11c,11d]^ In SIBs, the co‐intercalation of glyme molecules into the graphite lattice is highly reversible but also here ECD can give indications of side reactions, for example, in case the wrong conductive salt is used. **Figure** **5** shows results for two different electrolyte salts, NaOTf and NaTFSI. While a stable behavior is found for NaOTf, a continuous growth of the electrode is seen for NaTFSI. The continuous growth could be linked to continuous side reactions^[20i]^ and is a clear sign for issues related to the use of NaTFSI.

Another undesired process that can be detected by ECD is metal plating.^8^, ^9^, ^20^ Irreversible and reversible lithium plating has been observed for studies with pouch cells with the former leading to a continuous thickness increase.^8^, ^9^ ECD results on the insertion of sodium ions into hard carbon electrodes even indicate that sodium plating can take place already above 0 V (underpotential plating).^[20j]^ ECD can be also used to study additives for improved SEI formation on graphite electrodes. For lithium intercalation, Ivanov et al. found that the thickness of the SEI can be reduced by increasing the content of vinylene carbonate.^[11b]^ Vinylene carbonate is a well‐known co‐solvent that improves the quality of the SEI, leading to a more dense and hence thinner, structure.^[11b]^

### 2.4 Electrode Processes in Supercapacitors

**Figure 6:**
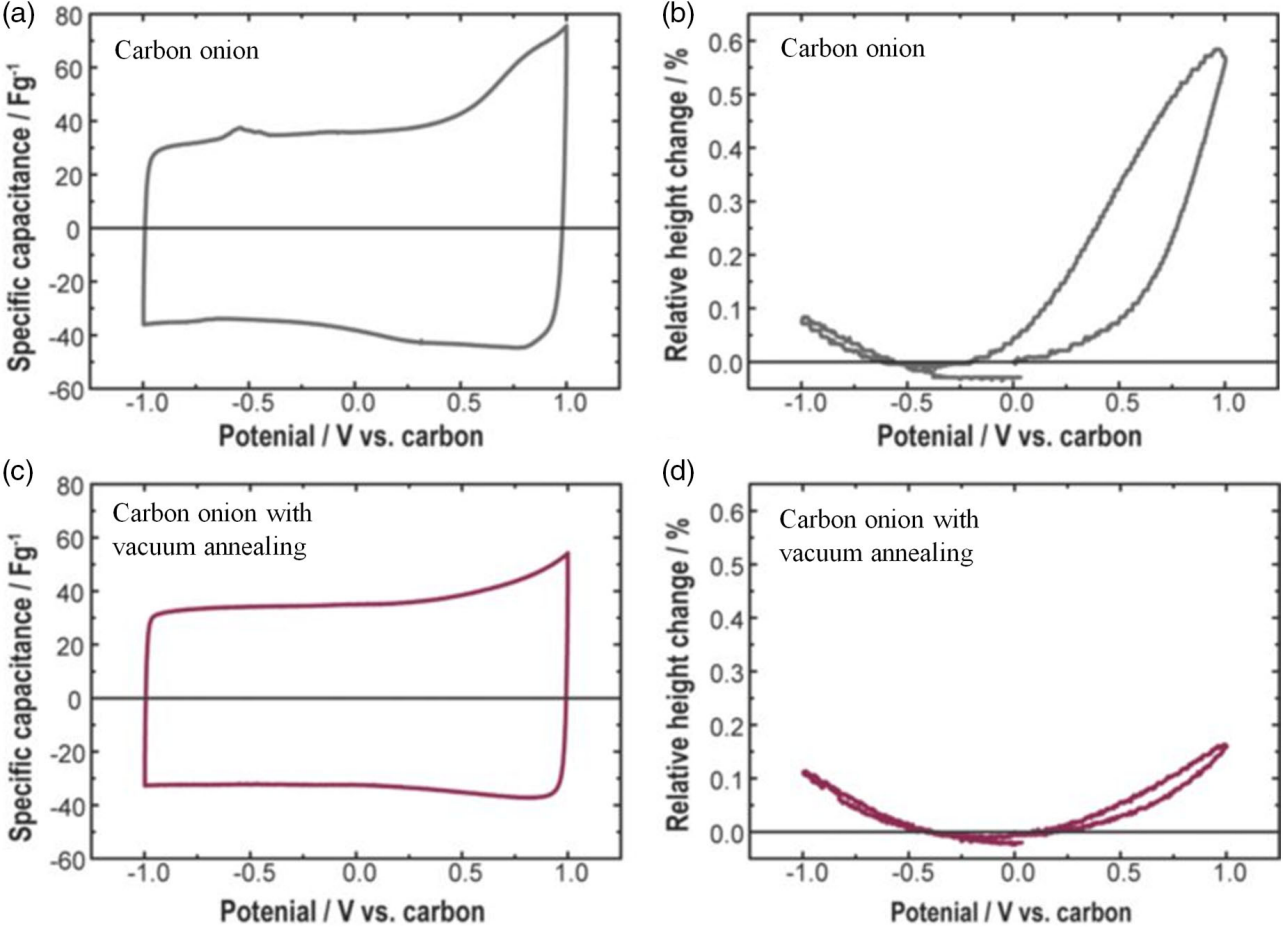
Cyclic voltammogram and thickness change (here named “height change”) of two different carbon electrodes with 1 M TEA‐BF_4_ in acetonitrile (sweep rate of 1 mV s^−1^). Adapted with permission.^[8l]^ Copyright 2012, The Electrochemical Society.

ECD has been also applied to supercapacitor electrodes. Only very small thickness changes are expected as long as only ion adsorption takes place.^8^, ^27^ Additional pseudocapacitive or intercalation processes lead to larger expansion.^8^, ^28^ Note that ECD research studies on supercapacitors typically use voltage sweep measurements (linear sweep voltammetry [LSV], cyclic voltammetry [CV]) rather than constant current measurements that are more popular in battery research. Hantel et al. showed that the thickness expansion of a carbon electrode (carbon onions) depends on their purity and the presence of functional groups. The higher thickness increase of the carbon onion sample during positive polarization (shown in **Figure** **6**b) can be explained by some minor Faradaic side reactions occurring due to metal impurities and the presence of functional groups. Vacuum annealing of the sample (see Figure 6d) results in higher carbon purity and therefore less undesired Faradic reactions occur, which translate into lower relative height changes.^[8l]^

**Figure 7:**
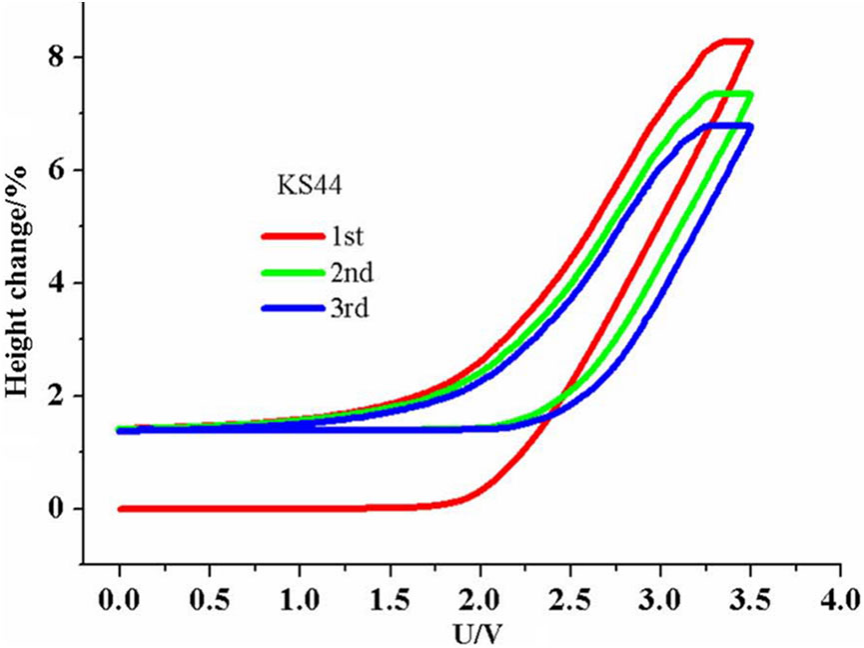
Change in electrode thickness (here named “height change”) versus voltage changes of a graphite/AC supercapacitor with SBPBF_4_ in propylene carbonate as electrolyte (SBP = spiro‐(1,1′)‐bipyrrolidinium). Reproduced with permission.^[28a]^ Copyright 2019, The Electrochemical Society.

A larger relative expansion is found when graphite is used as host material. The intercalation of BF_4_^−^ ions in an AC/graphite capacitor results in a thickness change of up to 8.25% (see **Figure** **7**).^[28a]^ Both publications proved that capacitive and pseudocapacitive processes can be distinguished using ECD in combination with CV.

Overall, ECD is a powerful method to obtain valuable information of electrode processes in batteries and supercapacitors. The technique has been successfully applied to study redox reactions, adsorption processes, side reactions, and SEI formation that lead to changes in the electrode thickness from the nanometer to the micrometer range. Compared with many other operando studies, several subsequent cycles can be recorded with ease, meaning that “activation cycles” of electrodes may be distinguished from stable cycling. Nevertheless, as it is a still a niche technique, no standardized measurement procedure as well as data evaluation has been established. The next section therefore provides a practical guideline for carrying out ECD measurements. The evaluation and presentation of ECD data as well as (undesired) factors that can influence the data are discussed.

## 3 Evaluation of ECD Data, Analysis, and Interpretation of the Data

ECD data can provide valuable information on various processes occurring at the electrodes. The following chapter includes an overview of the different characteristic thickness changes as well as their possible origins.

### 3.1 Data Presentation

**Figure 8:**
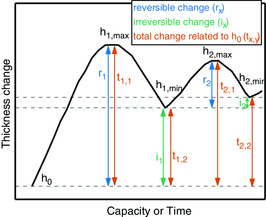
Typical ECD signal observed for negative electrodes (two full cycles at constant current). As the initial expansion is much larger compared with the following ones, the discussion of ECD results may require the definition of several parameters such as the initial electrode thickness (*h*_0_), the max./min. electrode expansion of a cycle *x* (*h*_*x,*max/min_), the reversible and irreversible changes within a cycle *x* (*r*_*x*_ *, i*_*x*_) or the total change in thickness related to *h*_0_. The figure is drawn in accordance with the literature.^8^, ^20^, ^22^

As no standardized data presentation of ECD has been established yet, care needs to be taken in comparing data from different literature sources. **Figure** **8** represents the most commonly used parameters to present and characterize the thickness change. The figure is drawn in accordance to the literature.^8^, ^20^, ^22^ Figure 8 represents the usual thickness change behavior of a negative electrode material, as those are the most common materials investigated by dilatometry (see Figure 4). The overall behavior for positive electrode materials may be different.

The reversible thickness change, also known as amplitude, represents the differences on the thickness between charge and discharge in one cycle and can be calculated using Equation (1). This thickness change is primarily influenced by the main reaction occurring in/at the electrode.

(1) $$r_{x}[\mu m]=h_{x,\text{max}}-h_{x,\text{min}}$$

The irreversible change, in contrast, is mainly attributed to side effects (e.g., side reactions, reorganization of the electrode particle network) and is the difference between the minimum value of one cycle compared with the previous one. It is determined using Equation (2).

(2) $$i_{x}[\mu m]=h_{x,\text{min}}-h_{x-1,\text{min}}$$

Finally, the total change represents the thickness change between the actual electrode thickness and the initial thickness. It has to be pointed out that the total change is not the sum of the reversible and irreversible thickness change. Herein, special focus lies on the difference between the totally lithiated or delithiated electrode and the initial thickness (see Equation (3) and (4)). Although the reversible change *r*_1_ and the total change *t*_1,1_ as well as the irreversible change *i*_1_ and the total change *t*_1,2_ are the same in the first cycle (see Figure 8), their values are different in the consecutive cycles.

(3) $$t_{x,1}[\mu m]=h_{x,\text{max}}-h_{0}$$

(4) $$t_{x,2}[\mu m]=h_{x,\text{min}}-h_{0}$$

Even though Equation ((1), (2), (3))–(4) exhibited the thickness change in absolute values (usually in micrometer), a presentation in relative values can be more convenient and easier for comparison, especially when two cells with different values for the initial electrode thicknesses are compared. Therefore, the different changes (all in micrometer ) can be referred to the initial thickness *h*_0_ or to the initial thickness of a specific cycle. A common way to follow the relative thickness change during cycling is shown in Equation 5, where *h*_n_ is the thickness of an electrode at each point.

(5) $$t_{n}[\%]=\frac{\left(h_{n}-h_{0}\right)}{h_{0}}*100\%$$

For a comparison of relative values between different publications, it is especially important to pay attention to how the values are calculated, as otherwise wrong numbers are compared.

Another important factor is how the initial value *h*_0_ is determined, especially when it is used as base value to calculate the relative thickness change. As an ECD setup is only capable of measuring thickness changes, the initial value needs to be measured ex situ, for example, with a digital gauge. While this appears simple at first, different values may be obtained depending on the initial state of the electrode. The dry and pristine electrode,^8^, ^9^ the electrode after soaking in electrolyte,^[14a]^ the electrode film excluding the current collector,^8^, ^9^, ^14^, ^22^ or the thickness after the equilibration time^8^, ^20^ have been used as reference point *h*_0_. There is no clear right or wrong for determining *h*_0_ as it depends on the experiment which method most suitable.

As all of the mentioned parameters strongly influence the data processing and graphical illustrations, it is crucially important that scientific studies need to clearly mention how the ECD data were processed. Particularly important is clear information on which base values were used when calculating relative changes and how the initial value *h*_0_ was determined.

**Figure 9:**
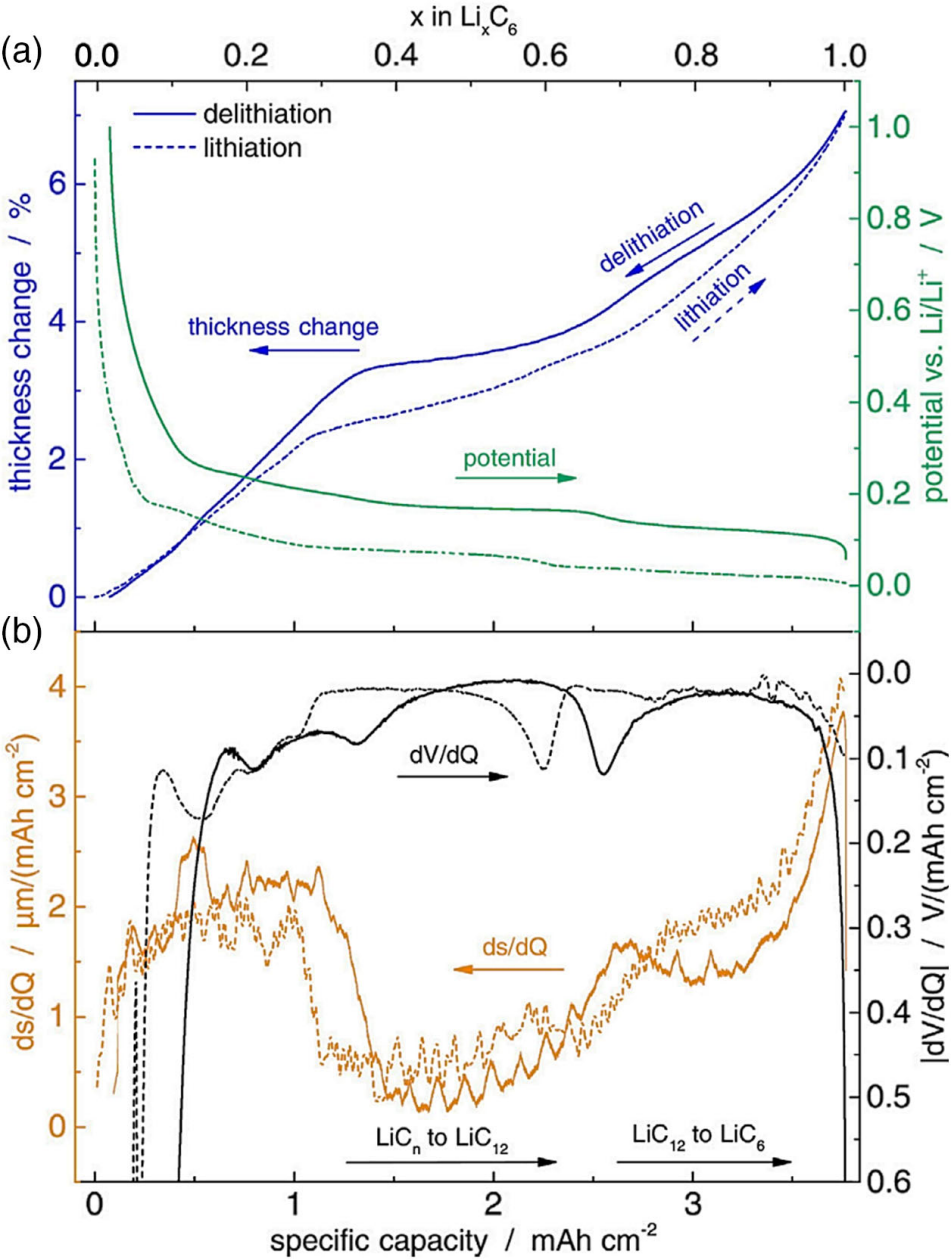
a) Thickness change and potential during the lithiation and delithiation of graphite electrodes. b) Differential thickness change and potential curve. Adapted with permission.^[9c]^ Copyright 2016, Elsevier Ltd.

Although the ECD experiment on batteries is usually conducted at constant current, that is, lithium is stored/released at a constant rate, the change in electrode thickness is not constant. This means that the rate at which the electrode expands/shrinks varies. Such a behavior can be more clearly seen from derivative plots (d*h*/d*t,*d*h*/d*Q*, or d^2^ *h*/d*Q* ^2^).^8^, ^9^, ^16^, ^18^, ^20^ Derivative plots can give insights into the storage mechanisms of ions taking place in the active material. For example, the well‐known step‐wise intercalation of lithium ions into graphite (LiC_*n*_ → LiC_12_ → LiC_6_)^[^ ^29^ ^]^ can be followed by ECD, and the derivative curve of the electrode expansion, which represents changes of the slope, can give information on the stages occurring at the specific state of charges (see **Figure** **9**).^[9c]^

In supercapacitors, a correlation between the orientation of the ions and the shape of the thickness change was found.^[27b]^ In addition, variations in the slopes can give information on the buffering ability of an electrode.^[14e]^

### 3.2 Interpretation of the Thickness Changes

This section discusses different mechanisms that cause variations of the electrode thickness. Obviously, the electrode material and the reaction occurring are the most relevant factors. As long as the electrode processes are reversible, battery and supercapacitor electrodes should show a reversible, periodic expansion/shrinkage over cycling. At the same time, a number of other factors can influence the measurement that can cause a more complex behavior. For example, side reactions, factors related to the electrode structure, and factors related to the measurement setup can have an influence. All factors are hard to disentangle, so care has to be taken when interpreting the thickness changes.

#### 3.2.1 Irreversible Expansion

The typical ECD data for negative electrode materials, see Figure 8 (*i*_1_ or *t*_1,2_), show an irreversible expansion in the first cycle. Several factors may cause such a behavior. One possible origin is the formation of the SEI, which is a well‐known process in LIBs.^11^, ^14^, ^16^, ^22^, ^30^ SEI formation is routed in the fact that negative electrodes operate outside the stability limit of the electrolyte solution. The initial lithiation of the electrode is therefore accompanied by electrolyte decomposition, which leads to the formation of a thin, protective surface film (SEI). This surface film prevents a continued electrolyte decomposition and therefore enables a long cycle life of the electrode. Note that the SEI is very thin (nanometer range) and hence at first cannot explain larger irreversible changes. However, the process of SEI formation also involves the formation of gases that might influence the thickness of the electrode (see Chapter 4 for gas evolution). Other explanations for the large irreversible change may be exfoliation,^8^, ^20^, ^31^ changes of the active material,^8^, ^11^, ^22^ swelling due to the electrolyte,^[22c]^ irreversible trapping of ions inside the structure,^[28b]^ or a more loose particle network.^8^, ^13^, ^32^

An increase in the electrode thickness during the following cycles (*i*_*n>1*_ > 0, see Figure 8) can be a sign for continuous SEI formation^8^, ^9^, ^14^ or for irreversible trapping of ions in the active material.^[8f]^ For both cases, the Coulomb efficiency (ratio between charge and discharge capacity) should be low as well. Other causes for continuous increase are changes on the electrode particles,^9^, ^11^ swelling of the electrode due to the electrolyte,^[14n]^ or a movement of the electrolyte between the sample and the piston from the sensor.^[11c,11d]^

#### 3.2.2 Irreversible Contraction

Even though an irreversible contraction in the first cycle seems to be less likely than an irreversible expansion, it can occur due to a collapse of the electrode structure, resulting from a loose connection between the different parts of the electrode (sudden decrease of the thickness)^[14e]^ or structural rearrangements.^[^ ^10^ ^]^ A continuous irreversible contraction might result from reorganization of the electrode material^14^, ^20^ or an irreversible loss of material.^[14j]^ The latter would also result in decreased capacity over time, which can be mitigated using a different binder material.^[14j]^

#### 3.2.3 Changes During Equilibration

The thickness change of the electrode should be also followed during the equilibration time, that is, prior to the actual test methods. Variation on this stage can occur due to dissolution of the active material in the electrolyte.^[22h]^ Sufficiently long equilibration should also mitigate the problem of possible electrode swelling during cycling (see also Chapter 4).

#### 3.2.4 Anisotropic Behavior of Charge/Discharge in Supercapacitors

Carrying out measurements with supercapacitors results in a thickness change on both electrodes due to the adsorption of anions and cations. In some cases, this thickness change appeared to be anisotropic. This can be explained by differences of the ion size. Herein, not the bare ion size has to be taken into account as the size of the unsolvated ion does not correlate with the measured thickness change.^8^, ^28^ Thus, the solvation of the ion might play an important role.^[8b,8i]^ In addition, some (co‐) intercalation reactions might take place on one electrode but not on the other.^[28d]^

## 4 Secondary Factors Influencing the Thickness Change

Section 3 addressed aspects of the ECD data with respect to the active material and how changes of the electrode thickness over cycling can be discussed. This section addresses additional, secondary factors that can be studied by ECD measurements, which need to be considered when comparing published data. These secondary factors can be classified into experimental factors relating to the electrode preparation (electrode porosity, loading, binder) and the electrolyte and instrumental factors that relate to the ECD setup and the electrochemical method used (constant current or voltage sweep experiment, temperature, applied pressure, gas evolution).

### 4.1 Electrode Porosity and Loading

**Figure 10:**
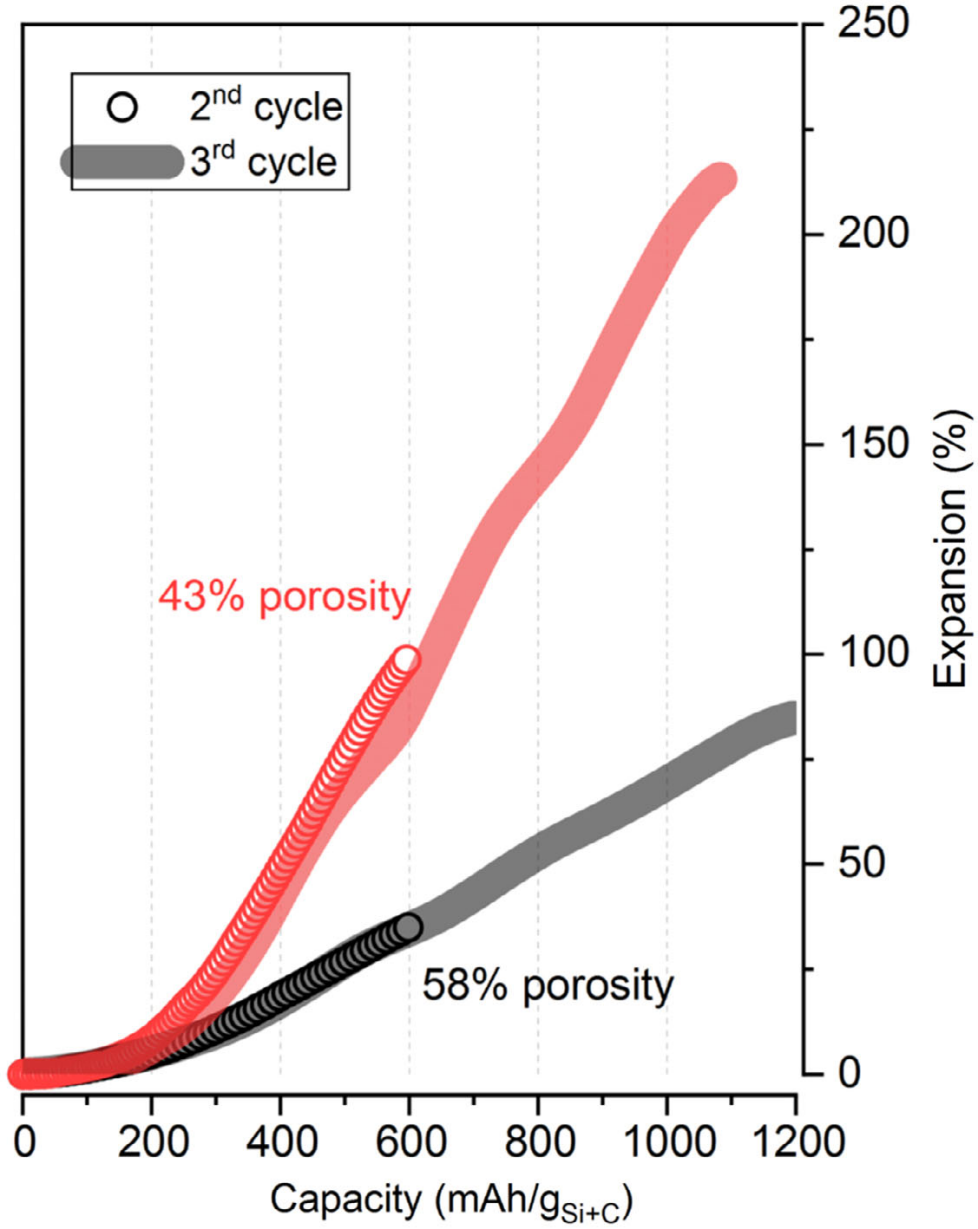
Expansion of a silicon/graphite electrode (30 wt% Si) during lithiation. Comparison of two different electrode porosities. Adapted under terms of the CC‐BY license.^[8a]^ Copyright 2020, the Authors. Published by IOP Publishing Limited.

The expansion/shrinkage of electrodes during cycling strongly depends on their porosity. A highly porous electrode can accommodate much of the materials’ volume changes within its pores. This means that the change in electrode thickness can be reduced by increasing the porosity.^8^, ^9^, ^14^, ^16^, ^20^, ^28^, ^33^ **Figure** **10** shows an example of such a buffering effect for two silicon/graphite electrodes with different porosities. Increasing the electrode porosity from 43% to 58% leads to a substantially smaller expansion of the electrode.^[8a]^

A high porosity is generally beneficial for obtaining better cycle life of battery electrodes that otherwise suffer from large volume changes. This explains the often excellent cycle life of many metal/carbon nanocomposite electrodes as they are usually highly porous, often >50%. Despite the advantage of achieving long cycle life with increasing porosity, the penalty is the low volumetric charge density of very porous electrodes and the additional weight as the pores are filled with electrolyte.^[20d]^ Commercial battery electrodes therefore show porosities in the range of only 20–30%. Buffering of volume changes in the electrode is often obtained using a second phase, for example, carbon materials^8^, ^12^, ^14^ or elastic polymer microspheres.^[14k]^

Another factor that influences the thickness change is the loading/thickness of an electrode.^11^, ^20^ It was found that higher loading results in higher (initial)^[20b]^ thickness change.^[11b]^ Overall, porosity and loading are important parameters influencing the degree of electrode breathing over cycling.

### 4.2 Binder

Electrodes typically contain some amount of binder that holds the electrode particles together and helps adhering them to the current collector foil. ECD can be used to study the effect of different binders on the electrode behavior, as shown by several publications.^8^, ^14^, ^20^, ^34^ Differences can be explained by several factors including different cohesive properties which might lead to the formation of cracks, delamination and destruction of the structure,^[14a]^ different available bonds which might result in particle rearrangement,^[14a]^ different mechanical properties such as their resistance to deformation^14^, ^35^ or elasticity^[14g]^ which can prevent the electrode from electrical disconnection during the electrode expansion,^[14j]^ and ability to uptake electrolyte (swelling) by different binders^[8c]^ or thermal properties of the binder, especially relevant during electrode preparation.^[9e]^ Different studies have been conducted comparing binder materials using ECD like polyvinylidene difluoride (PVDF),^8^, ^14^, ^15^, ^22^, ^34^ sodium salt of carboxymethyl cellulose (CMC),^8^, ^14^, ^22^, ^34^ CMC/styrene–butadiene rubber (SBR),^[8c]^ CMC/polyacrylic acid (PAA),^[14j]^ polyimide (PI),^14^, ^15^ lithiated polyacrylic acid (LiPAA),^[14a]^ polyvinyl alcohol (PVA),^[14a]^ or (poly(diallyldimethylammonium) bis(trifluromethan sulfonyl)imide) (PDDA).^[22h]^ Specified binder materials like crosslinked (CMC‐PAA)^14^, ^35^ or even dual cross linked (alginate grafted with polyacrylamide)^[8m]^ are designed and synthesized to accommodate the thickness change of electrodes with a high volume expansion. A limitation of many studies is that laboratory‐quality electrodes typically contain 10 wt% binder while the content is much lower for highly optimized commercial electrodes.

### 4.3 Electrolyte

The electrolyte can influence the thickness change of electrodes in various ways. Depending on its composition, SEI formation can be tailored and reversible solvent co‐intercalation may be realized. ECD can be helpful to identify effects of the electrolyte on the electrode behavior. For example, it can be used to differentiate between reactions with and without solvent co‐intercalation, as only the latter would lead to a large thickness change.^[20d]^ ECD can also help to understand rapid electrode fading in case poor electrolytes are chosen. For example, graphite electrodes show an unusual thickness expansion during lithium‐ion intercalation in case propylene carbonate is used as the electrolyte solvent^[11c,11d]^ or during sodium ion co‐intercalation in case NaTFSI is used as the electrolyte salt.^[20i]^ A potential limitation of using ECD for electrolyte studies is that ECD cells contain large amounts of excess electrolyte compared with commercial cells. In addition to the common liquid electrolytes, a few attempts have been made to use ECD for investigations on solid‐state batteries^[^ ^36^ ^]^ or batteries with gel or polymer electrolytes.^[^ ^37^ ^]^ As no stiff glass separator can be used in this case, the expansion of the whole cell rather than the electrode was measured.

### 4.4 Gas Evolution

The degree of measured gas evolution strongly depends on the electrode reactions that occur in the cell and whether the evolved gas can escape in a dead volume. Forcing the system to plate lithium results in higher gas evolution, seen by higher dilatation.^[9g]^ The application of external pressure to a pouch cell (spring or spindle) can reduce this influence and leads to lower thickness changes.^[9g]^ In commercial cells, the setup is designed in a way that the evolving gas can escape in a dead volume.^[8o]^ In addition, it was shown that a degassing step can help to achieve more reproducible data.^[8a]^

### 4.5 Electrochemical Technique

**Figure 11:**
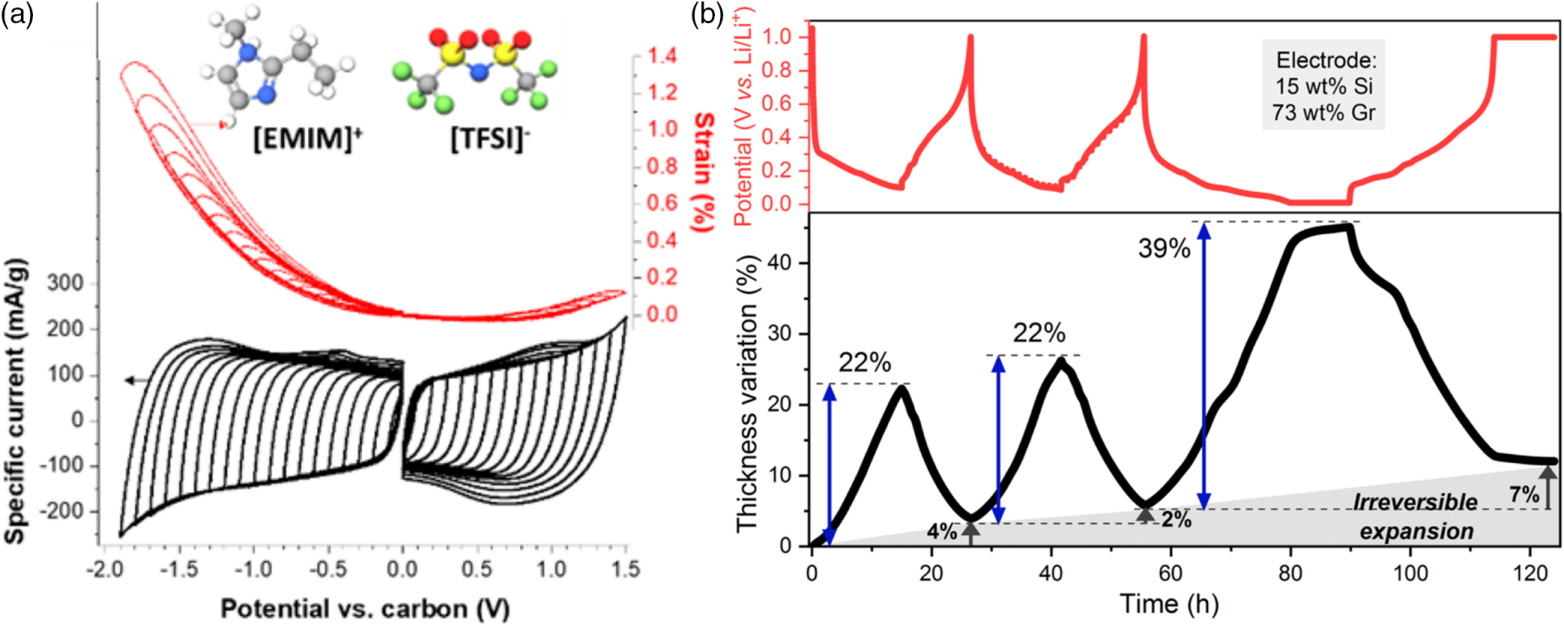
a) ECD using CV of carbon electrodes with EMIM‐TFSI electrolyte. Adapted with permission.^[27b]^ Copyright 2017, American Chemical Society. b) ECD using CC of the lithiation of a Si/graphite electrode. Reproduced under terms of the CC‐BY license.^[8a]^ Copyright 2020, the Authors. Published by IOP Publishing Limited.

The two main electrochemical techniques that are used with ECD are galvanostatic cycling (i.e., cycling at constant current, CC) and CV (i.e., cycling at a constant potential scan rate, CV). The first one is mainly used for batteries,^8^, ^9^, ^10^, ^11^, ^12^, ^13^, ^14^, ^15^, ^18^, ^20^, ^22^, ^30^ whereas the latter is commonly used for supercapacitors.^8^, ^27^, ^28^, ^33^, ^38^ Both types of measurements are compared in **Figure** **11**. The left image shows ECD results for a supercapacitor using CV. In this case, the voltage window is stepwise increased, which is a common variation of the CV method.^[27b]^ The right side shows EDC results for the lithiation of silicon/graphite electrode using a CC measurement.^[8a]^

A sufficiently small current or, respectively, scan rate should be applied in both methods to allow full utilization of the electrode. Too high currents and scan rates will only lead to partial electrode utilization and therefore only a fraction of the possible thickness change is measured. This can easily occur for ECD setups with a large distance between the electrodes which causes a larger ohmic drop compared with, for example, coin cells.^8^, ^9^, ^11^ For CV measurements, Kaasik et al. showed that the expansion for an infinite time (gained with chronoamperometric measurement) leads to an expansion up to 200% larger compared with when a scan rate of 5 mV s^−1^ is applied.^[27c]^ Similar results were also found by Hahn et al. where an increase in scan rate leads to a decrease in the thickness change.^[28d]^ In a similar way, small C rates are recommended to reach to the full volume expansion in batteries. In case the separator in ECD setups is large, high overpotentials may occur even at quite low C rates (*C*/8 for lithiation of graphite). One may therefore not reach the full electrode capacity and expansion during the measurement.^[11a]^ One may also apply an additional constant voltage step after charging/discharging to give the system additional time to reach to full capacity. An additional benefit of small C rates is the reduced heat development.^[16b]^

### 4.6 Equilibration Time

**Figure 12:**
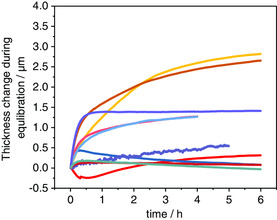
Thickness change during equilibration time for different ECD measurements (graphite and hard carbon electrodes in SIBs and LIBs) made in our lab. No electrochemical measurements were performed during this time. The thickness was set to zero for every electrode individually.

After assembly of the ECD setup, it is recommended to equilibrate the system to reach a stable starting condition. **Figure** **12** shows the thickness changes during the equilibration of various experiments collected from our lab. Despite using the same ECD device and climate chamber, there is no reproducible trend during equilibration. The different behavior could be due to the swelling of the binder material or different starting temperatures, but so far, we are unable to identify the relevant factors. The thickness changes range from a few tens of nanometers to few micrometers but they all seem to stabilize after several hours. Based on our experience, we recommend an equilibration step of at least 6 h while constantly monitoring the thickness change. From the graph, one can also see that the equilibration step can have an influence on the starting value for the initial electrode thickness *h*_0_ (see also Section 3.1).

### 4.7 Temperature

Temperature can have a strong effect on the ECD measurement. Two aspects have to be considered, first the internal temperature effect, emerging through self‐heating, and second the external temperature effect, caused by external change of temperature. Bitzer et al. performed a study where the thermal expansion through self‐heating was investigated by applying short charge/discharge pulses. Herein, if a change of the temperature of 1 K was measured on the cell surface, the thermal expansion was still smaller than the resolution of the device. Therefore, one can assume that no significant problem of self‐heating occurs when the temperature is kept constant at ±0.5 K.^[9g]^ Regarding the external temperature effect, Lee et al. investigated the influence of different surrounding temperatures and found that more side reactions occur at high temperatures, which lead to a thicker SEI and therefore a higher electrode expansion.^[9a]^ In addition, Spingler et al. pointed out the importance of constant temperature by measuring the thickness change at 25 °C and 35 °C. Even though 35 °C was favorable in their case as better ion transport through the thick separator was obtained, leading to less polarization, higher cell capacity, and a more stable displacement signal.^[22a]^ A thermal expansion of the cell can be due to various reasons. There is the actual thermal expansion of the materials (laminated film, current collector, separator, or electrode)^[17a]^ that always takes place. On the other hand, also electrolyte decomposition can take place at higher temperatures.^[20f]^ In any case, ECD measurements should be conducted in climate chambers to minimize any external temperature effects.

### 4.8 Applied Pressure

Even though a spring is used in most of ECD setups, the pressure/force on the electrode is still lower compared with traditional coin cells or Swagelok‐type cells^8^, ^14^ (≈1 N for commercial ECDs^14^, ^38^ and 30 N estimated for Swagelok‐type cells^[14d]^ or ≈20 kPa for commercial ECDs and ≈185 kPa for coin cells^8^, ^39^). This reduced pressure can result in higher irreversible capacities as well as a more rapid capacity decay due to delamination and contact loss between the active material, the conductive additive, and the current collector.^8^, ^14^ In this sense, it was found that the thickness change is different between ECD and Swagelok cells or coins cells, although no general trend can be seen.^[14c,14h]^

### 4.9 Position of the Sensor

The position of the sensor is also a variable that influences the measured thickness change. Considering especially pouch cells, no standard and preferential position can be found as the best place always depends on the specific cell.^[9g]^ However, it was found that the thickness change measured on the edges of the cell provided misleading information, as, for example, reduced expansion^9^, ^16^ or plating processes^9^, ^40^ were found here. In addition, lateral expansions could also be translated into vertical expansions at the edges, if the electrode has a similar size than the cell body.^[11a]^ However, it was found that nearly the whole change on the unit‐cell level results in an increase in thickness, as the length and width are nearly infinite compared with the thickness of the electrode.^[9c]^ In addition, the in‐plane expansion is prohibited by the current collector.^[14e]^ Other factors that might be different over the area of the electrode, and therefore be influenced by the position of the sensor, might be different states of charge or different local thermal expansions.^[9c]^

### 4.10 Sensor Drift/Air Leakage

The sensor drift is a characteristic of the used device (e.g., <20 nm h^−1^ for ECD‐3 nano from El‐Cell^[8o]^) and is especially important for long‐time experiments.^[8a]^ This problem can be overcome using a drift correction or focusing on qualitative instead of quantitative comparisons.^[14c]^ A possible cause for the sensor drift may be also air leakage. Air leakage is an important point that does not only influence the sensor drift but might also have an impact on the electrode expansion. As ECD cells are not as easily sealed as, for example, coin cells, air or moisture may enter the cell over time, leading to side reactions with the electrodes or the electrolytes (e.g., formation of HF due to hydrolysis of the conductive salt LiFP_6_), and therefore influence the electrode expansion.^[8a]^ However, the degree to which air leakage influences the ECD measurement strongly depends on the individual measurement (how well the cell is sealed) as well as cell chemistry being studied (how reactive the components are).

## 5 Comparison of ECD with XRD

When studying the storage behavior of battery electrodes during cycling, the method of choice is usually X‐ray diffraction (XRD) as direct information on the structure changes of the active material is obtained. In contrast to this, ECD (Type 1) measures thickness changes of the electrode, which means that it takes also other (in)active components and morphological characteristics of the electrode into account. Moreover, ECD is also sensitive to changes in materials with large disorder such as hard^[20c,20j]^ or soft^[28b]^ carbon, which are difficult to study by XRD. From that point of view, both techniques complement each other very well and they generally show similar qualitative trends for expansion/shrinkage during cycling.^9^, ^28^ Ideally, both methods are applied in operando mode though this is more difficult to implement for XRD.

**Figure 13:**
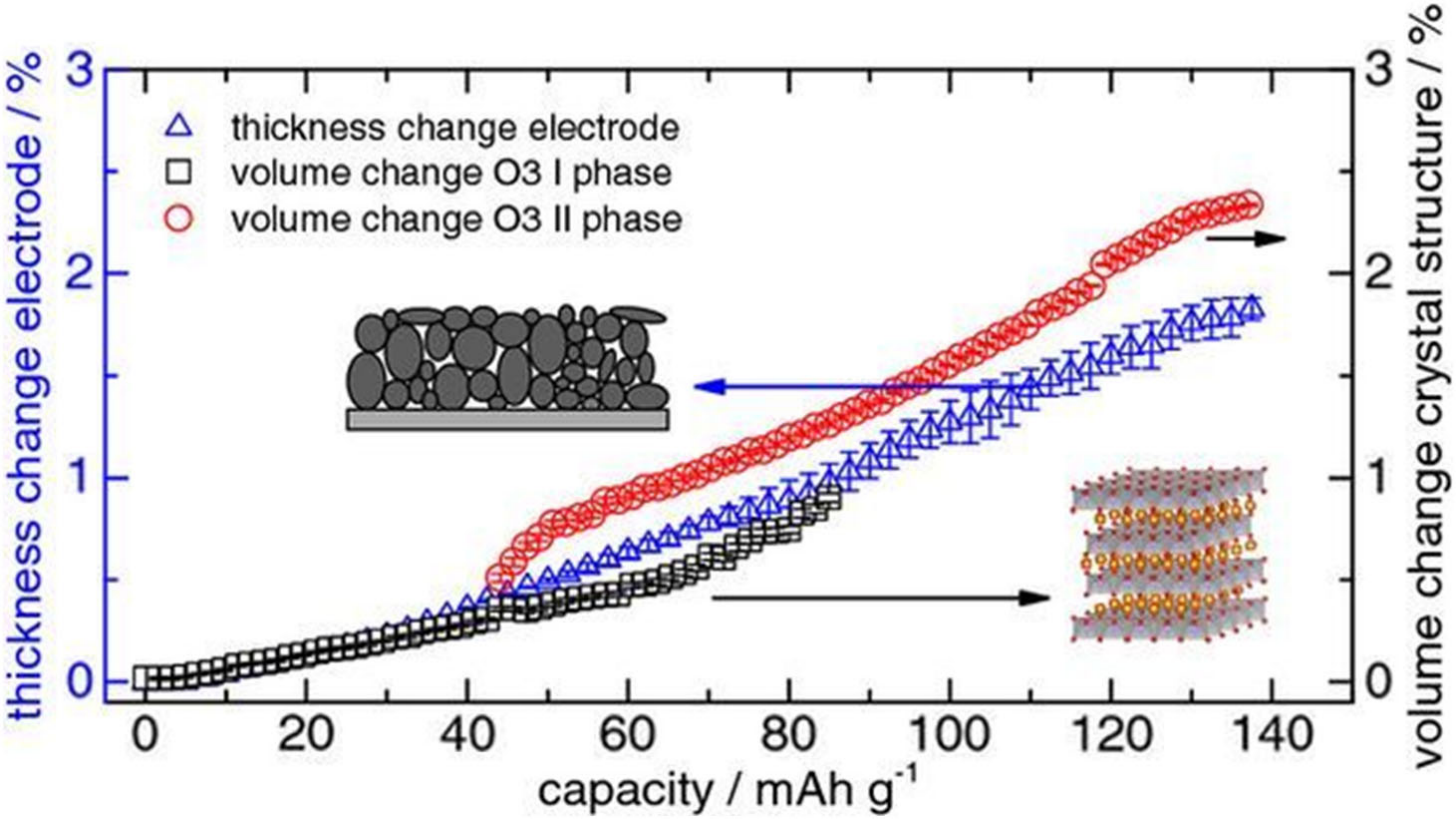
Comparison of the changes of the lattice unit‐cell parameters (measured by XRD) and the thickness change of the electrode (measured by ECD) during the delithiation of a lithium cobalt oxide (LCO) electrode. Adapted with permission.^[^ ^25^ ^]^ Copyright 2016, The Electrochemical Society.

Various studies have been published that contain a combined analysis of XRD and ECD data. Rieger at all studied the expansion of a commercial LiCoO_2_ material by operando XRD and dilatometry.^[^ ^25^ ^]^ The intercalation of lithium ions results in the expansion of its crystal structure and consequently also of the electrode. **Figure** **13** shows the changes in crystal volume and electrode thickness, as determined by operando XRD and ECD, respectively. Both signals correlate well although the change by XRD (2.32%) is slightly larger compared with what is found by ECD (1.8%). This might be explained by the buffering phenomena inside the electrode as the electrode exhibits a porosity of around 33%.^[^ ^25^ ^]^

**Figure 14:**
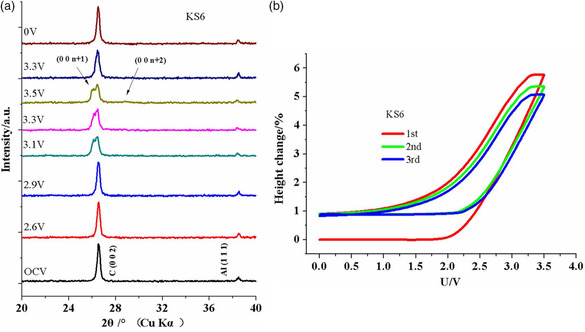
Intercalation of BF_4_^−^ ions in a graphite/AC capacitor. a) XRD studies at different states of charge. b) ECD measurement. Reproduced with permission.^[28a]^ Copyright 2019, The Electrochemical Society.

Li et al. studied the intercalation of BF_4_^−^ into graphite in an AC/graphite hybrid supercapacitor; see **Figure** **14**. The intercalation was done using a CV measurement. While changes in the diffraction patterns could be found starting from 3.1 V, the ECD method indicated that electrode processes already started from 1.8 V.^[28a]^

**Figure 15:**
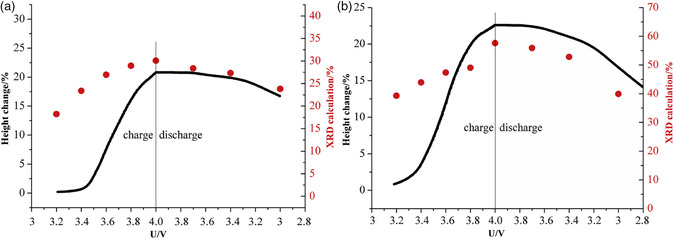
Comparison of the height change calculated from XRD and the height change measured with ECD of the graphitized mesocarbon microbead electrodes in a supercapacitor with 1.5 m SBPBF_4_ in PC as electrolyte: a) positive electrode and b) negative electrode. Adapted with permission.^[28b]^ Copyright 2018, Elsevier Ltd.

In another study on supercapacitors, Li et al. studied graphitized mesocarbon microbeads as electrode with 1.5 m SBPBF_4_ (SBP = spiro‐(1,1′)‐bipyrrolidinium) in propylene carbonate (PC) as electrolyte.^[28b]^ They compared the height change measured by ECD to the change of the crystal structure measured with in situ XRD for both electrodes during the first cycle; see **Figure** **15**. While both techniques showed similar results during discharging, clear differences appeared during charging. This is explained by the expansion of the graphite particles in different directions, as well as restrictions from the binder or the conductive additive in the beginning. At a higher voltage, the changes of the crystal structure are more directly transferred into a thickness change.^[28b]^

The observation that the electrode expansion measured by ECD is smaller compared with what is expected from XRD is not unusual and has been frequently reported in literatures.^8^, ^9^, ^10^, ^25^, ^28^, ^33^ Various parameters, especially the electrode porosity, account for this difference; see Section 4.1. In addition, it was found that for a comparison with ECD values in some cases it is better to use the lattice parameter “*c*” than the unit cell volume.^16^, ^22^

## 6 Conclusion and Future Trends

Operando ECD is a powerful technique to study the behavior of batteries and supercapacitors during charging and discharging. The method can easily track dimensional changes from the nanometer to the micrometer range and can therefore be used to follow various processes, including electrode reactions, surface reactions like SEI formation, or side reactions. A major focus in the last years was on studying the expansion and shrinkage (breathing) of electrode materials for LIBs and SIBs. Herein, the effects of different parameters like the binder material, the composition of the electrolyte, or the porosity of the electrode have been investigated. Detailed analysis of the thickness change (e.g., derivative plots) can give also information on the storage mechanism, similar to XRD. Both methods are complementary and ECD can provide information when the use of XRD is less successful, for example, in case of disordered materials. Moreover, XRD measures crystallographic properties while ECD measures the whole electrode, therefore, it includes effects of real electrodes such as the porosity. Even though the number of ECD studies increased in the past years, it can still be considered as a relatively new technique. No standard measurement protocol has been therefore established yet. This review gave an overview on how to present and discuss ECD data and the kinds of factors influencing the experimental results. This includes secondary effects such as the porosity of the electrode material (a major factor), the used binder, the electrolyte, gas evolution, or the electrochemical measurement itself, as well as the equilibration time. It is also highlighted that a clearer description of the data evaluation is needed to ensure comparability between different research studies. This especially relates to how the initial electrode thickness is defined.

Important trends for further improvement of ECD relate to the applied pressure and the stability of the device. With the commercial dilatometer from EL‐Cell, the ECD‐3, the electrode is only loaded with a low and constant force of ≈1 Newton per cm^2^ during the measurement. This is the minimum force considered necessary to contact the electrode reliably. Higher forces have the disadvantage that the electrode shows stronger creep. In many cases, however, it can be useful and relevant for the practice to increase the force by orders of magnitude and also measure the force during the cycle by integrating additional actuators and sensors. This is especially true for solid‐state battery electrodes. Modulation of the force can also be interesting, for example, to investigate the (visco‐)elastic behavior of the electrodes, analogous to the established dynamic mechanical analysis (DMA) technique. In addition to the general improvement of the resolution and accuracy of the expansion measurement, a very general goal for the further development of ECD is, as with all operando techniques, to make the electrochemical stability as good as that of the real battery. The use of improved sealing concepts such as glass‐to‐metal seals and the exclusive use of battery‐grade construction materials points the way here. This will open the door for true long‐term measurements.

## Conflict of Interest

The authors declare no conflict of interest.

## Supporting information

Supplementary Material
