# Supplementary material for: A Practical Guide for Using Electrochemical Dilatometry as Operando Tool in Battery and Supercapacitor Research
Source: Energy Technol (Weinh). 2022 Mar 10;10(5):2101120. doi: 10.1002/ente.202101120 (PMC9285449; doi:10.1002/ente.202101120)
Supplement: Supplementary file 1 — Supplementary Material [file ENTE-10-0-s001.pdf]

Supporting information

A practical guide for using electrochemical dilatometry as operando tool in battery and supercapacitor research

Ines Escher[a], Dr. Matthias Hahn[b] , Dr. Guillermo A. Ferrero[a], Prof. Philipp Adelhelm[a,c]*

a Humboldt Universität zu Berlin, Institut für Chemie, Brook-Taylor-Str. 2, 12489 Berlin, Germany

b EL-Cell GmbH, Tempowerkring 8, 21079 Hamburg, Germany

c Helmholtz-Zentrum Berlin, Joint research group Operando Battery Analysis (CE-GOBA), Hahn-Meitner-Platz 1, 14109 Berlin, Germany

E-mail: philipp.adelhelm@hu-berlin.de

Table 1. Comparison of the thickness change in Lithium-Ion Batteries in the first cycle for different electrode materials. The thickness of the de-intercalated state is used as starting value. Please be aware that the values might be just approximations, as they are gained out of the graphs in the original paper.

**Lithium-Ion Batteries**			
Material	electrode side	$t_{1,1}$ [%]	reference
**Si related samples**			
**Si thin film**			
**Si (with and without PI capping)**	neg. electrode	340<br>275	[1]
**Si (with and without artificial LIPON SEI)**	neg. electrode	693<br>443	[2]
**Si**	neg. electrode	140	[3]
**Si + other metals**			
**Si-Te-Fe-Al alloy<br>(different temperature treatment)**	neg. electrode	531<br>312<br>436	[4]
**Ti/Si multilayer film**	neg. electrode	3<br>16	[3]
**Zn/Si multilayer film**	neg. electrode	4.5<br>31	[3]
**Al/Si multilayer film**	neg. electrode	16<br>31	[3]

**Si (alloy) (different binder)**	neg. electrode	490<br>230<br>169	[5]
**Si + carbon**			
**Si + graphite**	neg. electrode	22	[6]
**Si (influence of slurry pH)**	neg. electrode	170 +- 40	[7]
**Si (binder comparison)**	neg. electrode	450<br>410<br>300	[8]
**Si (different conductive additives)**	neg. electrode	410<br>85	[9]
**Si (different binder)**	neg. electrode	212<br>201<br>145	[10]
**Si (different particle sizes)**	neg. electrode	350<br>140	[11]
**Si + graphite (different binder, different kind of graphite)**	neg. electrode	6.4<br>8<br>5.4<br>8.5	[12]
**Si-C**<br>**Nano particles or yolk-shell nano particles**	neg. electrode	90<br>10	[13]
**Si (bare Si or Si-encapsulating hollow carbon)**	neg. electrode	30<br>18	[14]
**Si (nanosized Si with cellulose fibers)**	neg. electrode	47.5	[15]
**Si-embedded carbon nanotubes (different amount of CNTs)**	neg. electrode	110<br>125<br>210<br>215<br>280	[16]
**Si + carbon nanoplatelets (calendered electrode)**	neg. electrode	258	[17]
**Si + graphite (different binder)**	neg. electrode	97<br>204<br>326<br>451	[18]
**SiO**			
**SiO/C (with and without PI coating)**	neg. electrode	158<br>65	[19]
**SiO**	neg. electrode	275	[20]
**Carbon**			
**Graphite**			
**Graphite**	neg. electrode	4.5<br>8.5	[21]
**Graphite (graphite from commercial cell)**	neg. electrode	7	[22]
**Graphite**	neg. electrode	4.25	[23]
**Graphite (different graphites)**	neg. electrode	4.3<br>5.3<br>6.0	[24]
**Graphite (different graphites and binder)**	neg. electrode	3.7<br>6.75<br>2.5<br>4.5	[12]

**Graphite (calendered and non-calendered)**	neg. electrode	6<br>12.5	[25]
**Graphite co-intercalation**			
**Graphite (co-intercalation)<br>(different binder)**	neg. electrode	175<br>142	[26]
**Hard cabon**			
**Hard carbon<br>(different binder)**	neg. electrode	4.5<br>2	[27]
**Zn related samples**			
**ZnO + carbon<br>(carbon physically mixed or as matrix)**	neg. electrode	36<br>25	[28]
**Sn related samples**			
**Li alloy (Sn, SnSb)**	neg. electrode	55<br>40	[29]
**SnO_2 (with and without PI capping)**	neg. electrode	175<br>250	[30]
**Mn related samples**			
**MnS<br>(with and without S-doped carbonaceous mesoporous framework)**	neg. electrode	11.3<br>34.1	[31]
**Ge related samples**			
**$Ge/Cu_3Ge/C$**	neg. electrode	68	[32]
**Ge/C**	neg. electrode	147	[32]
**Sb related samples**			
**Microsized Sb particles (different amount of PI coating)**	neg. electrode	110<br>40<br>52	[33]
**Sb**	neg. electrode	110	[34]
**Sb_2Se_3**	neg. electrode	50	[34]
**Sb_2S_3**	neg. electrode	80	[34]
**Fe related samples**			
**Fe_3O_4/reduced GO**	neg. electrode	23.6	[35]
**Fe_2O_3 / C**	neg. electrode	44	[36]
**$Fe_2O_3/Fe/C$**	neg. electrode	86	[36]
**Sulfur related samples**			
**Li_2S**	pos. electrode	0.5	[37]
**S (dissolution of sulfur)<br>(different binder)**	pos. electrode	-13<br>-25<br>-35	[38]
**NMC**			
**NMC**	pos. electrode	0.7	[39]
**NCA**			
**NCA**	pos. electrode	0.8	[25]
**LCO**			
**LCO**	pos. electrode	-1.6	[22]

**Graphite (anion intercalation)**			
**Graphite (anion intercalation) (different binder and different electrochemical measurements)**	pos. electrode	20.9<br>32.7<br>124.5<br>82.3<br>38.1<br>33.8<br>73.3	[40]
**Graphite (anion intercalation) (different anions intercalated)**	pos. electrode	64.8<br>30.1	[41]

Table 2. Comparison of the thickness change in Sodium-Ion Batteries in the first cycle for different electrode materials. The thickness of the de-intercalated state is used as starting value. Please be aware that the values might be just approximations, as they are gained out of the graphs in the original paper.

**Sodium-Ion Batteries**			
Material	electrode side	$t_{1,1}$ [%]	reference
**Carbon**			
**Graphite**			
**Graphite**	neg. electrode	190	[42]
**Graphite (different salts)**	neg. electrode	144<br>100	[43]
**Graphite (different electrolytes)**	neg. electrode	135<br>109<br>114<br>96<br>99<br>88<br>132	[44]
**Graphite (different binder)**	neg. electrode	146<br>175<br>118<br>140<br>142	[26]
**Graphite nanoplatelets**			
**GnP (graphite nanoplatelets)**	neg. electrode	19.5	[45]
**Hard carbon**			
**Hard carbon (different pyrolysis temperatures)**	neg. electrode	0.75<br>2.3<br>2.6<br>2.4<br>2.3<br>2.6	[46]
**Hard carbon (different binder)**	neg. electrode	6.5<br>1	[27]

**<u>Sn related samples</u>**			
**Sn_4P_3 (+ NHC)<br>(with and without Nitrogen doped hard carbon)**	neg. electrode	31<br>53	[47]
**Sn_4P_3**	neg. electrode	165	[48]
**SnNGnP<br>(Sn at nitrogen-doped graphite nanoplatelets)**	neg. electrode	7.8	[45]
**SnSb<br>(different synthesis procedure)**	neg. electrode	33<br>74	[49]
**Sn+Sb<br>(different synthesis procedure)**	neg. electrode	40<br>48	[49]
**SntGraphite<br>(Sn and graphite)**	neg. electrode	124	[50]

References:

1. Lee, P.-K.; Tahmasebi, M. H.; Tan, T.; Ran, S.; Boles, S. T.; Yu, D. Y. W., Polyimide capping layer on improving electrochemical stability of silicon thin-film for Li-ion batteries. *Materials Today Energy* **2019,** *12*, 297-302.
2. Jiménez, A. R.; Nölle, R.; Wagner, R.; Hüsker, J.; Kolek, M.; Schmuch, R.; Winter, M.; Placke, T., A step towards understanding the beneficial influence of a LIPON-based artificial SEI on silicon thin film anodes in lithium-ion batteries. *Nanoscale* **2018,** *10* (4), 2128-2137.
3. Pan, H.; Zhang, J.; Chen, Y.; Zhuo, X.; Yang, Y., In-situ dilatometric study of Metal/Si multilayer film electrodes. *Thin Solid Films* **2010,** *519* (2), 778-783.
4. Park, H. I.; Lee, D. G.; Chung, D. J.; Sohn, M.; Park, C.; Kim, H., Real-Time Dilation Observation of Si-Alloy Electrode Using Thermally Treated Poly (Amide-Imide) as a Binder for Lithium Ion Battery. *Bulletin of the Korean Chemical Society* **2019,** *40* (3), 248-253.
5. Yoon, D.-H.; Marinaro, M.; Axmann, P.; Wohlfahrt-Mehrens, M., Study of the Binder Influence on Expansion/Contraction Behavior of Silicon Alloy Negative Electrodes for Lithium-Ion Batteries. *J. Electrochem. Soc.* **2020,** *167* (16), 160537.
6. Prado, A. Y. R.; Rodrigues, M.-T. F.; Trask, S. E.; Shaw, L.; Abraham, D. P., Electrochemical Dilatometry of Si-bearing Electrodes: Dimensional Changes and Experiment Design. *J. Electrochem. Soc.* **2020,** *167*.
7. Tranchot, A.; Idrissi, H.; Thivel, P. X.; Roué, L., Impact of the slurry pH on the expansion/contraction behavior of silicon/carbon/carboxymethylcellulose electrodes for Li-ion batteries. *J. Electrochem. Soc.* **2016,** *163* (6), A1020.
8. Yu, D. Y. W.; Zhao, M.; Hoster, H. E., Suppressing vertical displacement of lithiated silicon particles in high volumetric capacity battery electrodes. *ChemElectroChem* **2015,** *2* (8), 1090-1095.
9. Karkar, Z.; Mazouzi, D.; Hernandez, C. R.; Guyomard, D.; Roué, L.; Lestriez, B., Threshold-like dependence of silicon-based electrode performance on active mass loading and nature of carbon conductive additive. *Electrochim. Acta* **2016,** *215*, 276-288.
10. Kim, J. S.; Choi, W.; Cho, K. Y.; Byun, D.; Lim, J.; Lee, J. K., Effect of polyimide binder on electrochemical characteristics of surface-modified silicon anode for lithium ion batteries. *J. Power Sources* **2013,** *244*, 521-526.
11. Tranchot, A.; Idrissi, H.; Thivel, P.-X.; Roué, L., Influence of the Si particle size on the mechanical stability of Si-based electrodes evaluated by in-operando dilatometry and acoustic emission. *J. Power Sources* **2016,** *330*, 253-260.
12. Gómez-Cámer, J. L.; Bünzli, C.; Hantel, M. M.; Poux, T.; Novák, P., On the correlation between electrode expansion and cycling stability of graphite/Si electrodes for Li-ion batteries. *Carbon* **2016,** *105*, 42-51.

13. Xiao, X.; Zhou, W.; Kim, Y.; Ryu, I.; Gu, M.; Wang, C.; Liu, G.; Liu, Z.; Gao, H., Regulated breathing effect of silicon negative electrode for dramatically enhanced performance of Li-Ion battery. *Adv. Funct. Mater.* **2015,** *25* (9), 1426-1433.
14. Park, Y.; Choi, N. S.; Park, S.; Woo, S. H.; Sim, S.; Jang, B. Y.; Oh, S. M.; Park, S.; Cho, J.; Lee, K. T., Si-encapsulating hollow carbon electrodes via electroless etching for lithium-ion batteries. *Adv. Energy Mater.* **2013,** *3* (2), 206-212.
15. Gómez Cámer, J. L.; Morales, J.; Sánchez, L.; Ruch, P.; Ng, S. H.; Kötz, R.; Novák, P., Nanosized Si/cellulose fiber/carbon composites as high capacity anodes for lithium-ion batteries: A galvanostatic and dilatometric study. *Electrochim. Acta* **2009,** *54* (26), 6713-6717.
16. Park, S.; Kim, T.; Oh, S. M., Electrochemical dilatometry study on Si-embedded carbon nanotube powder electrodes. *Electrochem Solid St* **2007,** *10* (6), A142-A145.
17. Karkar, Z.; Jaouhari, T.; Tranchot, A.; Mazouzi, D.; Guyomard, D.; Lestriez, B.; Roué, L., How silicon electrodes can be calendered without altering their mechanical strength and cycle life. *J. Power Sources* **2017,** *371*, 136-147.
18. Gendensuren, B.; Oh, E.-S., Dual-crosslinked network binder of alginate with polyacrylamide for silicon/graphite anodes of lithium ion battery. *J. Power Sources* **2018,** *384*, 379-386.
19. Tan, T.; Lee, P.-K.; Zettsu, N.; Teshima, K.; Yu, D. Y. W., Highly stable lithium-ion battery anode with polyimide coating anchored onto micron-size silicon monoxide via self-assembled monolayer. *J. Power Sources* **2020,** *453*, 227874.
20. Kim, T.; Park, S.; Oh, S. M., Solid-state NMR and electrochemical dilatometry study on Li+ uptake/extraction mechanism in SiO electrode. *J. Electrochem. Soc.* **2007,** *154* (12), A1112-A1117.
21. Michael, H.; Iacoviello, F.; Heenan, T. M. M.; Llewellyn, A.; Weaving, J. S.; Jervis, R.; Brett, D. J. L.; Shearing, P. R., A Dilatometric Study of Graphite Electrodes during Cycling with X-ray Computed Tomography. *J. Electrochem. Soc.* **2021,** *168* (1), 010507.
22. Rieger, B.; Schlueter, S.; Erhard, S. V.; Schmalz, J.; Reinhart, G.; Jossen, A., Multi-scale investigation of thickness changes in a commercial pouch type lithium-ion battery. *Journal of Energy Storage* **2016,** *6*, 213-221.
23. Bauer, M.; Wachtler, M.; Stöwe, H.; Persson, J. V.; Danzer, M. A., Understanding the dilation and dilation relaxation behavior of graphite-based lithium-ion cells. *J. Power Sources* **2016,** *317*, 93-102.
24. Hahn, M.; Buqa, H.; Ruch, P. W.; Goers, D.; Spahr, M. E.; Ufheil, J.; Novák, P.; Kötz, R., A dilatometric study of lithium intercalation into powder-type graphite electrodes. *Electrochem. Solid-State Lett.* **2008,** *11* (9), A151-A154.
25. Spingler, F. B.; Kücher, S.; Phillips, R.; Moyassari, E.; Jossen, A., Electrochemically Stable In Situ Dilatometry of NMC, NCA and Graphite Electrodes for Lithium-Ion Cells Compared to XRD Measurements. *J. Electrochem. Soc.* **2021,** *168* (4), 040515.
26. Escher, I.; Kravets, Y.; A. Ferrero, G.; Goktas, M.; Adelhelm, P., Strategies for Alleviating Electrode Expansion of Graphite Electrodes in Sodium-ion Batteries Followed by In Situ Electrochemical Dilatometry. *Energy Technology* **2020,** *9*, 2000880.
27. Escher, I.; A. Ferrero, G.; Goktas, M.; Adelhelm, P., In Situ (Operando) Electrochemical Dilatometry as a Method to Distinguish Charge Storage Mechanisms and Metal Plating Processes for Sodium and Lithium Ions in Hard Carbon Battery Electrodes. *Advanced Materials Interfaces* **2021**, 2100596.
28. Chae, O. B.; Park, S.; Ryu, J. H.; Oh, S. M., Performance Improvement of Nano-Sized Zinc Oxide Electrode by Embedding in Carbon Matrix for Lithium-Ion Batteries. *J. Electrochem. Soc.* **2013,** *160* (1), A11-A14.
29. Besenhard, J. O.; Yang, J.; Winter, M., Will advanced lithium-alloy anodes have a chance in lithium-ion batteries? *J. Power Sources* **1997,** *68* (1), 87-90.
30. Li, Y.; Wang, S.; Lee, P.-K.; He, J.; Yu, D. Y. W., Crack-resistant polyimide coating for high-capacity battery anodes. *J. Power Sources* **2017,** *366*, 226-232.

31. Ma, Y.; Ma, Y.; Kim, G. T.; Diemant, T.; Behm, R. J.; Geiger, D.; Kaiser, U.; Varzi, A.; Passerini, S., Superior Lithium Storage Capacity of α-MnS Nanoparticles Embedded in S-Doped Carbonaceous Mesoporous Frameworks. *Adv. Energy Mater.* **2019,** *9* (43), 1902077.
32. Chae, O. B.; Park, S.; Ku, J. H.; Ryu, J. H.; Oh, S. M., Nano-scale uniform distribution of Ge/Cu3Ge phase and its electrochemical performance for lithium-ion batteries. *Electrochim. Acta* **2010,** *55* (8), 2894-2900.
33. Wang, S.; Lee, P.-K.; Yang, X.; Rogach, A. L.; Armstrong, A. R.; Yu, D. Y. W., Polyimide-cellulose interaction in Sb anode enables fast charging lithium-ion battery application. *Materials today energy* **2018,** *9*, 295-302.
34. Wang, S.; Yang, X.; Lee, P.-K.; Rogach, A. L.; Yu, D. Y. W., Reversible interaction of Sb with an active Se matrix enhances the cycle stability of electrodes for lithium-ion batteries. *Chemistry of Materials* **2019,** *31* (7), 2469-2475.
35. Kim, H.-K.; Roh, K. C.; Kim, K.-B., In situ electrochemical dilatometric study of Fe3O4/reduced graphene oxide nanocomposites as anode material for lithium ion batteries. *J. Electrochem. Soc.* **2015,** *162* (12), A2308.
36. Kim, J.; Chung, M. K.; Ka, B. H.; Ku, J. H.; Park, S.; Ryu, J.; Oh, S. M., The role of metallic Fe and carbon matrix in Fe2O3/Fe/carbon nanocomposite for lithium-ion batteries. *J. Electrochem. Soc.* **2010,** *157* (4), A412-A417.
37. Li, M.; Wang, Z.; Detsi, E., In Situ Electrochemical Dilatometry Study of (De) lithiation and Polysulfide Dissolution-Induced Dimensional Changes in Lithium-Sulfur Cathodes during Charging and Discharging. *J. Electrochem. Soc.* **2020,** *167* (5), 050505.
38. Lemarié, Q.; Idrissi, H.; Maire, E.; Thivel, P.-X.; Alloin, F.; Roué, L., Impact of the binder nature on the morphological change of sulfur electrodes upon cycling investigated by in situ characterization methods. *J. Power Sources* **2020,** *477*, 228374.
39. Nayak, P. K.; Yang, L.; Pollok, K.; Langenhorst, F.; Aurbach, D.; Adelhelm, P., Investigation of Li1. 17Ni0. 20Mn0. 53Co0. 10O2 as an Interesting Li-and Mn-Rich Layered Oxide Cathode Material through Electrochemistry, Microscopy, and In Situ Electrochemical Dilatometry. *ChemElectroChem* **2019,** *6* (10), 2812-2819.
40. Huesker, J.; Froböse, L.; Kwade, A.; Winter, M.; Placke, T., In situ dilatometric study of the binder influence on the electrochemical intercalation of bis (trifluoromethanesulfonyl) imide anions into graphite. *Electrochim. Acta* **2017,** *257*, 423-435.
41. Huesker, J.; Winter, M.; Placke, T., Dilatometric study of the electrochemical intercalation of bis (trifluoromethanesulfonyl) imide and hexafluorophosphate anions into carbon-based positive electrodes. *ECS Transactions* **2015,** *69* (22), 9.
42. Goktas, M.; Bolli, C.; Berg, E. J.; Novák, P.; Pollok, K.; Langenhorst, F.; Roeder, M. v.; Lenchuk, O.; Mollenhauer, D.; Adelhelm, P., Graphite as Cointercalation Electrode for Sodium-Ion Batteries: Electrode Dynamics and the Missing Solid Electrolyte Interphase (SEI). *Adv. Energy Mater.* **2018,** *8* (16), 1702724.
43. Goktas, M.; Bolli, C.; Buchheim, J.; Berg, E. J.; Novák, P.; Bonilla, F.; Rojo, T.; Komaba, S.; Kubota, K.; Adelhelm, P., Stable and Unstable Diglyme-Based Electrolytes for Batteries with Sodium or Graphite as Electrode. *ACS Appl. Mater. Interfaces* **2019,** *11* (36), 32844-32855.
44. Karimi, N.; Varzi, A.; Passerini, S., A comprehensive insight into the volumetric response of graphite electrodes upon sodium co-intercalation in ether-based electrolytes. *Electrochim. Acta* **2019,** *304*, 474-486.
45. Palaniselvam, T.; Goktas, M.; Anothumakkool, B.; Sun, Y.-N.; Schmuch, R.; Zhao, L.; Han, B.-H.; Winter, M.; Adelhelm, P., Sodium Storage and Electrode Dynamics of Tin-Carbon Composite Electrodes from Bulk Precursors for Sodium-Ion Batteries. *Adv. Funct. Mater.* **2019,** *29* (18), 1900790.
46. Alptekin, H.; Au, H.; Jensen, A. C.; Olsson, E.; Goktas, M.; Headen, T. F.; Adelhelm, P.; Cai, Q.; Drew, A. J.; Titirici, M.-M., Sodium Storage Mechanism Investigations through Structural Changes in Hard Carbons. *ACS Applied Energy Materials* **2020,** *3* (10), 9918-9927.
47. Palaniselvam, T.; Mukundan, C.; Hasa, I.; Santhosha, A. L.; Goktas, M.; Moon, H.; Ruttert, M.; Schmuch, R.; Pollok, K.; Langenhorst, F.; Winter, M.; Passerini, S.; Adelhelm, P., Assessment on

the Use of High Capacity "Sn4P3"/NHC Composite Electrodes for Sodium-Ion Batteries with Ether and Carbonate Electrolytes. *Adv. Funct. Mater.* **2020,** *30* (42), 2004798.
48. Wang, W.; Zhang, J.; Yu, D. Y. W.; Li, Q., Improving the cycling stability of Sn4P3 anode for sodium-ion battery. *J. Power Sources* **2017,** *364*, 420-425.
49. Brehm, W.; Buchheim, J. R.; Adelhelm, P., Reactive and Nonreactive Ball Milling of Tin-Antimony (Sn-Sb) Composites and Their Use as Electrodes for Sodium-Ion Batteries with Glyme Electrolyte. *Energy Technology* **2019,** *7* (10), 1900389.
50. Palaniselvam, T.; Babu, B.; Moon, H.; Hasa, I.; Santhosha, A. L.; Goktas, M.; Sun, Y.-N.; Zhao, L.; Han, B.-H.; Passerini, S.; Balducci, A.; Adelhelm, P., Tin-Containing Graphite for Sodium-Ion Batteries and Hybrid Capacitors. *Batteries & Supercaps* **2021,** *4* (1), 173-182.
